# Identification of potential biomarkers of inflammation-related genes for ischemic cardiomyopathy

**DOI:** 10.3389/fcvm.2022.972274

**Published:** 2022-08-23

**Authors:** Jianru Wang, Shiyang Xie, Yanling Cheng, Xiaohui Li, Jian Chen, Mingjun Zhu

**Affiliations:** ^1^Department of Cardiovascular, The First Affiliated Hospital of Henan University of Chinese Medicine, Zhengzhou, China; ^2^Central Laboratory, The First Affiliated Hospital of Henan University of Chinese Medicine, Zhengzhou, China; ^3^Department of Vascular Disease, Shanghai TCM-Integrated Hospital, Shanghai University of Traditional Chinese Medicine, Shanghai, China; ^4^Institute of Vascular Anomalies, Shanghai Academy of Traditional Chinese Medicine, Shanghai, China

**Keywords:** ischemic cardiomyopathy, inflammation-related genes, biomarker, nomogram, bioinformatics analyses, heart failure

## Abstract

**Objective:**

Inflammation plays an important role in the pathophysiology of ischemic cardiomyopathy (ICM). We aimed to identify potential biomarkers of inflammation-related genes for ICM and build a model based on the potential biomarkers for the diagnosis of ICM.

**Materials and methods:**

The microarray datasets and RNA-Sequencing datasets of human ICM were downloaded from the Gene Expression Omnibus database. We integrated 8 microarray datasets *via* the SVA package to screen the differentially expressed genes (DEGs) between ICM and non-failing control samples, then the differentially expressed inflammation-related genes (DEIRGs) were identified. The least absolute shrinkage and selection operator, support vector machine recursive feature elimination, and random forest were utilized to screen the potential diagnostic biomarkers from the DEIRGs. The potential biomarkers were validated in the RNA-Sequencing datasets and the functional experiment of the ICM rat, respectively. A nomogram was established based on the potential biomarkers and evaluated *via* the area under the receiver operating characteristic curve (AUC), calibration curve, decision curve analysis (DCA), and Clinical impact curve (CIC).

**Results:**

64 DEGs and 19 DEIRGs were identified, respectively. 5 potential biomarkers (SERPINA3, FCN3, PTN, CD163, and SCUBE2) were ultimately selected. The validation results showed that each of these five potential biomarkers showed good discriminant power for ICM, and their expression trends were consistent with the bioinformatics results. The results of AUC, calibration curve, DCA, and CIC showed that the nomogram demonstrated good performance, calibration, and clinical utility.

**Conclusion:**

SERPINA3, FCN3, PTN, CD163, and SCUBE2 were identified as potential biomarkers associated with the inflammatory response to ICM. The proposed nomogram could potentially provide clinicians with a helpful tool to the diagnosis and treatment of ICM from an inflammatory perspective.

## Introduction

Heart failure (HF) is generally considered a complex clinical syndrome with symptoms and/or signs caused by structural and/or functional cardiac abnormalities, leading to considerable morbidity and mortality (1). Data from 2020 showed that there were an estimated 23 million HF patients worldwide, with a 1-year mortality rate of 7.2% and a 5-year survival rate of 25% after hospitalization (2). There were 12.1 million patients with HF and 3.0 million patients with incident HF ≥ 25 years old in China (3). HF imposes a significant burden on global health systems. Therefore, it is socially important to actively study countermeasures to prevent and treat HF.

Over the past few decades, the aetiologies of HF have shifted from valvular heart disease and hypertension to coronary artery disease (4). The term ischemic cardiomyopathy (ICM) is also known as ischemic heart disease in many cases and manifests as the syndrome of HF due to chronic left ventricle systolic dysfunction resulting from underlying coronary artery disease (4, 5). ICM is the most common but underestimated manifestation and cause of HF and also the main cause of mortality in patients with HF. The evidence from clinical, angiographic, and autoptic findings demonstrates a more complex pathophysiological process in ICM (6). Although massive studies have been made in exploring the pathological mechanisms of ICM, it remains poorly understood. There is no doubt that an in-depth exploration of the pathological mechanisms of ICM will bring novel insights and ideas for its diagnosis and treatment.

Inflammation plays an important role in the pathophysiology of many cardiovascular diseases. Strong correlations were observed between the elevated level of inflammation and the several stages of ICM (7). Clinically, there are some limitations in determining the severity of inflammation in ICM. In recent decades, the studies on biomarkers of cardiovascular disease and their clinical application have increased exponentially (8). As is well known, early diagnosis is extremely important for effective treatment and prognosis of the disease. Several inflammation-related factors including C-reactive protein, interleukin (IL) 6, galectin-3, etc., are considered biomarkers for ICM (9). However, the limited number of important and specific inflammatory biomarkers for ICM has become a growing problem in its diagnosis and treatment. Against this background, it is significant to optimize the diagnosis and treatment of ICM from the perspective of inflammation.

With the continuous development and popularization of high-throughput technologies such as biochips and second-generation sequencing, data information such as transcriptomics and epigenetics of many disease pathological processes have been accessed by researchers, and these massive data provide important support for researchers to deeply explore and reveal the mechanisms and patterns of the occurrence, development, and regression of human diseases. Machine learning (ML), a subset of artificial intelligence, has been used in several medical fields (10, 11). ML algorithms can discover complex patterns and powerful evidence in large volumes of medical data to support clinical decision-making (10–12). There is a trend that multiple ML algorithms such as least absolute shrinkage and selection operator (LASSO), support vector machine recursive feature elimination (SVM-RFE), random forest (RF) are used to screen disease biomarkers and therapeutic targets, explore pathogenesis and predict clinical outcomes, which will allow for more rigorous and standardized processes. Many studies have also attempted to explore the possible pathological progression mechanisms of ICM by combining data from the public database, bioinformatics analysis, and ML algorithms (13–15). However, few studies have been conducted to identify potential inflammation-related diagnostic genes for ICM. Hence, it is very important to explore the immune-related diagnostic biomarkers that can make the early diagnosis of ICM possible.

**FIGURE 1:**
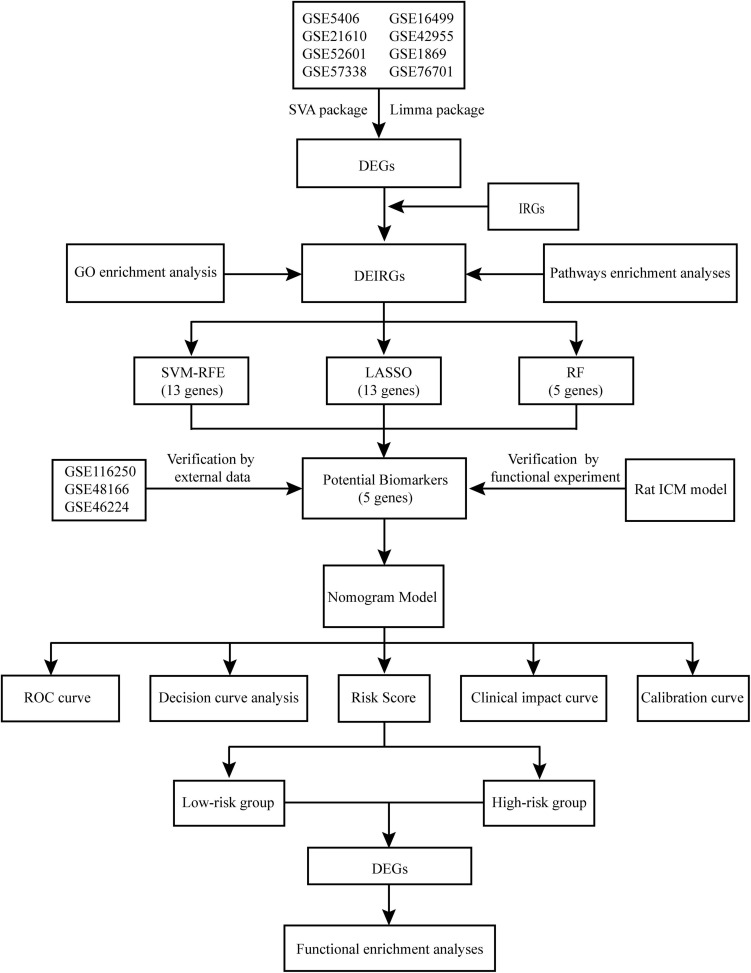
Flowchart of the study design.

In this study, we integrated 8 microarray datasets to screen the differentially expressed genes (DEGs) between ICM and non-failing control (NFC) myocardial tissue. Then, the differentially expressed inflammation-related genes (DEIRGs) were identified by intersecting the inflammation-related genes (IRGs) with DEGs. Subsequently, three ML algorithms were used to screen the promising diagnostic biomarkers of ICM from DEIRGs. The expression levels of these potential biomarkers were validated in additional RNA-Sequencing datasets and the functional experiment of the ICM rat. Finally, a nomogram model based on the potential biomarkers was established to predict ICM (Figure 1). We hope that our results can further strengthen the understanding of the role of inflammation in ICM and contribute to the development of promising diagnostic and therapeutic strategies.

## Materials and methods

### Data acquisition and preprocessing

The ICM-associated datasets were extracted from the Gene Expression Omnibus (GEO) database.^1^ The relevant information of the datasets included in the current study were shown in Supplementary Table 1A. The research population in this study included 315 patients with ICM and 232 subjects in the NFC group. The characteristics of the population were listed in Supplementary Table 1B. The probe names in the microarray datasets were converted to corresponding gene symbols using Perl script. The microarray datasets, GSE5406, GSE16499, GSE21610, GSE42955, GSE52601, GSE1869, GSE57338, and GSE76701, were merged into one dataset (the merged dataset) and the batch effects among microarrays were removed using the SVA package (16). The RNA-Sequencing datasets, GSE116250, GSE48166, and GSE46224, were used as external validation datasets.

### Identification and functional enrichment analyses of differentially expressed inflammation-related genes

The DEGs between NFC and ICM samples in the merged dataset were screened by limma package (17) with the threshold criteria of | log_2_ fold change (FC)| > 0.585 and *P*-adj < 0.05. The IRGs were extracted from DisGeNET^2^ and the Molecular Signature database.^3^ 467 IRGs were obtained from the DisGeNET. 4 gene sets (M5932, M15877, M13807, and M38152) were downloaded from the MSigDB database. Ultimately, 1746 IRGs were yielded after eliminating the duplicates. DEIRGs were identified by intersecting the IRGs with DEGs identified in the merged dataset. The functional enrichment analyses of DEIRGs were performed in Metascape^4^ (18), using Gene ontology (GO), KEGG Pathway, Reactome Gene Sets, and WikiPathways. *P* < 0.01, a minimum count of 3, and the enrichment factor > 1.5 were set as the thresholds.

### Screening potential biomarkers based on machine learning

We used LASSO *via* glmnet package (19, 20), SVM-RFE *via* e1071 package (21), and RF *via* “randomForest” package (22) to screen vital biomarkers for ICM from DEIRGs, respectively. The ML algorithms parameters were set as follows: LASSO, cvfit = cv.glmnet (nfold = 10, family = “binomial,” type.measure = “class”); SVM = rfe (functions = caretFuncs, method = “cv,” methods = “svmRadial”); randomForest (ntree = 500). Characteristic genes with the minimum cross-validation error were used as output files. The potential diagnostic biomarkers were yielded by intersecting the vital biomarkers identified by three algorithms. The diagnostic value of the biomarker was evaluated by the receiver operating curves (ROC) curve in the merged dataset. Concurrently, their expressions and diagnostic values were also validated in the external validation datasets.

### Validation of the biomarkers expressions in the rat ischemic cardiomyopathy model

Male SD rats aged 6–8 weeks (purchased from Beijing Weitong Lihua Experimental Animal Technology Co. Ltd., production license: SCXK (Jing)-2016-0011) were randomly divided into sham and ICM groups (*n* = 6/group). The rats were fed under a 12 h cycle of light/dark in IVC condition and had free access to food and water. A rat ICM model was constructed by permanent ligating the left anterior descending coronary artery, as previously described (23). At 8 weeks, M-mode echocardiography in the left ventricular parasternal long-axis view was performed using a Vivid E9 (GE Vingmed, Horten, Norway). Serum B-type natriuretic peptide (BNP) levels in sham and ICM rats were measured with rat BNP ELISA kits (Elabscience Biotechnology, China). Meanwhile, wheat germ agglutinin (WGA, Sigma, L4895) staining was performed to evaluate the size of the cardiomyocytes, as previously described (24). The myocardial pathological changes were also detected by conventional hematoxylin-eosin (HE) staining kit (G1120, Solarbio, Beijing, China) and Masson staining kit (G1340, Solarbio, Beijing, China). Proteins were extracted from ischemic left ventricular regions of rats. After separation *via* SDS-PAGE, the proteins were transferred to the PVDF membranes (Millipore, Darmstadt, Germany). The membranes were blocked with 5% BSA and incubated with the primary antibodies at 4°C overnight. Then the secondary antibody was added and incubated for 1 h. Finally, the blots were visualized using the ECL Plus kit (Solarbio, PE0010, Beijing, China) and exposed to a ChemiDoc MP Imaging System (Bio-Rad, CA, United States). Protein density was measured using Image Lab software. The variation of protein density was expressed as fold changes compared to the sham in the blot. The antibodies used in this study were as follows: CD163 (Proteintech, Cat#16646-1-AP; 1:600), PTN (Immunoway, Cat#YT5519; 1:1000), FCN3 (Immunoway, Cat#YN2366; 1:1000), SCUBE2 (Abcepta, Cat#ALS13965; 1:500), SERPINA3 (Immunoway, Cat#YT5391; 1:1000), GAPDH (Immunoway, Cat#YM3029; 1:10000).

### Establishment and evaluation of a nomogram model

The “rms” package was employed to build a nomogram model based on the potential biomarkers selected by three ML algorithms. The calibration curve and ROC curve were employed to estimate the predictive power of the nomogram model. Decision curve analysis (DCA) and clinical impact curve (CIC) were used to evaluate the clinical value of the nomogram model. The risk score for each sample was derived from the nomogram model.

### Functional enrichment analyses of the differentially expressed genes grouped by risk score

All samples in the merged dataset were dichotomized into the low-risk and high-risk groups according to the median risk score. The DEGs between the low-risk and high-risk groups were screened by limma package with the cutoff criteria of | log_2_ FC| > 0.585 and *P*-adj < 0.05. The functional enrichment analyses of DEGs were implemented in Metascape *via* the reference gene set and screening conditions mentioned above.

### Statistical analysis

R software (version 4.1.0) was used in our study. The data were shown as mean ± SD and analyzed using IBM SPSS statistics 21.0 software. Student’s *t*-test or Wilcoxon test was used to compare the data between the 2 groups. A *P* value < 0.05 was accepted as statistically significant.

## Results

### Screening of differentially expressed inflammation-related genes in the merged dataset

**FIGURE 2:**
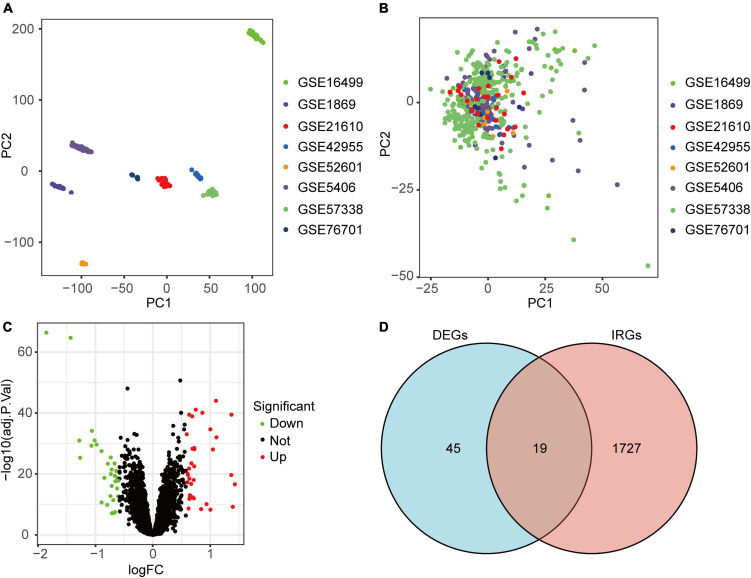
Identification of DEIRGs in ICM. **(A)** Two-dimensional principal component analysis cluster plot before merging of 8 datasets *via* SVA package. **(B)** Two-dimensional principal component analysis cluster plot after merging of 8 datasets *via* SVA package. **(C)** Volcano plot of the DEGs in the merged dataset. **(D)** Venn diagram of 19 DEIRGs shared by 64 DEGs and 1746 IRGs.

In our research, 8 microarray datasets were merged into a merged dataset, which contained 194 NFC and 270 ICM samples. Figures 2A,B indicated that batch-to-batch variation was removed. According to the screening method and criteria mentioned above, a total of 64 DEGs between NFC and ICM samples in the merged dataset were obtained, including 34 up-regulated and 30 down-regulated genes (Figure 2C). Finally, we obtained 19 DEIRGs by crossing the 64 DEGs with 1746 IRGs (Figure 2D).

### Functional enrichment analyses of differentially expressed inflammation-related genes

We performed functional enrichment analyses to elucidate the roles of the DEIRGs during ICM. 75 biological processes, 9 cellular components, and 15 molecular functions were identified. We enriched 3, 20, and 11 pathways from the KEGG, Reactome, and Wiki databases, respectively. By manually curation, the GO terms and pathways that had an adjusted *p*-value of below 0.05 were shown in Supplementary Tables 2, 3. The significantly enriched GO terms related to ICM included inflammatory response, cell chemotaxis, extracellular matrix, antioxidant activity, and so forth. The pathways closely associated with ICM were mainly the apelin signaling pathway, toll-like receptors (TLRs) cascades, IL-18 and IL-17 signaling pathway, neutrophil degranulation, and so forth.

### Screening potential diagnostic biomarkers for ischemic cardiomyopathy

**FIGURE 3:**
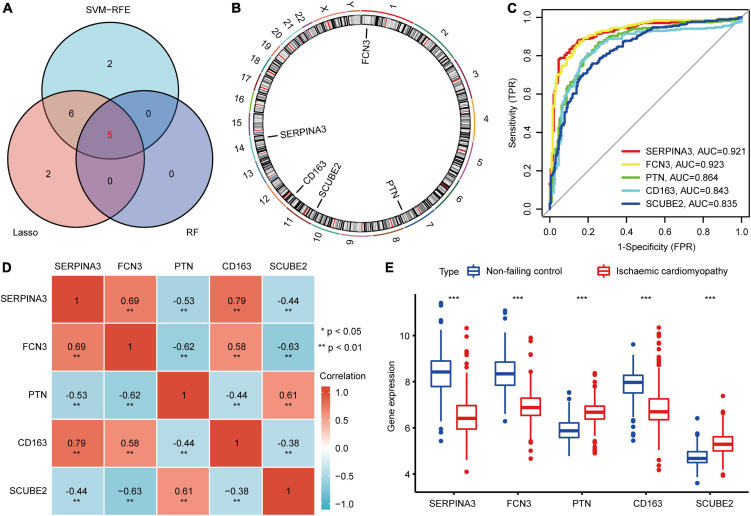
Identification of potential diagnostic biomarkers for ICM. **(A)** Venn diagram of the biomarkers extracted from LASSO, RF, and SVM-RFE algorithms. **(B)** The chromosomal positions of the 5 biomarkers. **(C)** ROC curve of the diagnostic effectiveness of the biomarkers. **(D)** Heatmap of correlations between the 5 biomarkers. **(E)** The differential expression histogram of the 5 biomarkers between the NFC and ICM samples from the merged dataset. ***P < 0.001.

We identified 13 genes from the DEIRGs as biomarkers for ICM using the LASSO algorithm under lambda.min = 0.0021 (Supplementary Figures 1A,B). Meanwhile, 13 DEIRGs were recognized as vital biomarkers using the SVM-RFE algorithm (Supplementary Figure 1C). The RF algorithm was adopted to rank the importance of the DEIRGs according to MeanDecreaseGini (Supplementary Figures 1D,E). The top 5 DEIRGs based on MeanDecreaseGini values were used as important biomarkers for subsequent analysis. To obtain the robust potential biomarkers in ICM, the vital biomarkers from three ML algorithms were overlapped. 5 potential biomarkers were ultimately selected (Figure 3A), including SERPINA3, FCN3, PTN, CD163, and SCUBE2. Figure 3B showed the chromosomal positions of the 5 potential biomarkers. A powerful diagnostic capacity was confirmed in the merged dataset with an AUC of 0.921 (95% CI 0.895–0.947) in SERPINA3, AUC of 0.923 (95% CI 0.898–0.948) in FCN3, AUC of 0.864 (95% CI 0.829–0.899) in PTN, AUC of 0.843 (95% CI 0.804–0.882) in CD163, and AUC of 0.835 (95% CI 0.798–0.872) in SCUBE2 (Figure 3C). Correlation between biomarkers was assessed using Spearman correlation analysis. There was a significant positive correlation between SERPINA3 and CD163, and a significant negative correlation between FCN3 and SCUBE2 (Figure 3D). Moreover, the expression levels of SERPINA3, FCN3, and CD163 were lower in ICM samples compared with NFC samples in the merged dataset, while the opposite was true for PTN and SCUBE2 (Figure 3E).

### External validation of the potential biomarkers in the external validation datasets

To obtain more accurate and reliable results, we performed ROC analysis and verified the expression levels of 5 potential biomarkers in the three external validation datasets, respectively. The results showed that their expression trends in the external validation datasets were consistent with those in the merged dataset (Supplementary Figures 2A–C), and they also had good discriminant power in distinguishing between NFC and ICM samples, as evidenced by AUC > 0.6 (Supplementary Figures 2D–F).

### Further validation of the biomarkers in the rat ischemic cardiomyopathy model

**FIGURE 4:**
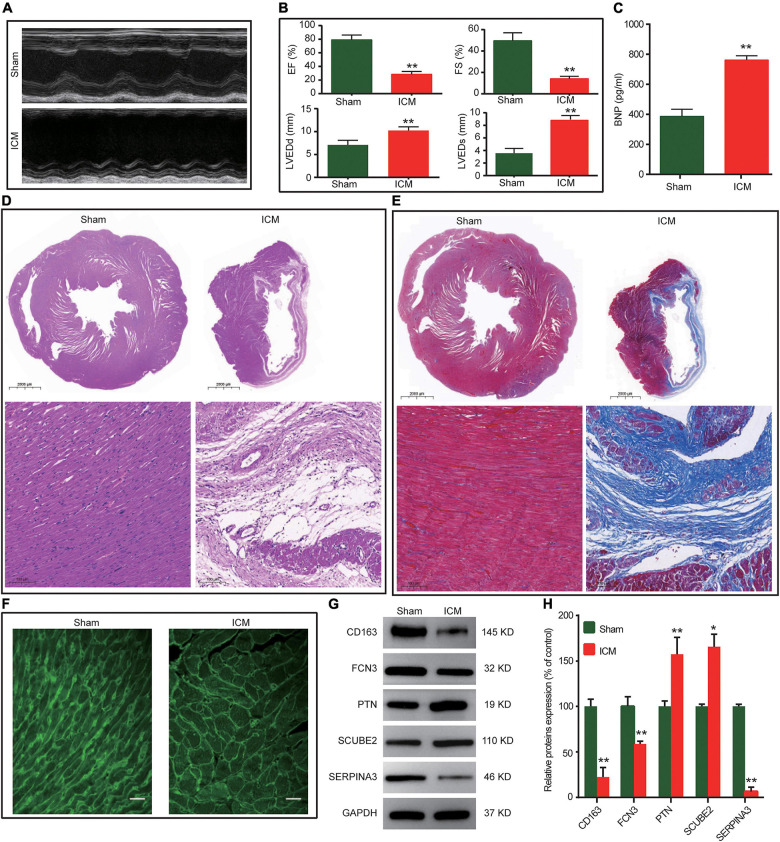
The validation of the biomarkers in the rat ICM model. **(A)** Representative M-mode echocardiographic images for each group. **(B)** Echocardiographic parameters for each group. (*n* = 6). **(C)** Serum BNP levels by ELISA (*n* = 6). **(D)** Representative images of HE staining. **(E)** Representative images of Masson staining. **(F)** Representative images of WGA staining. **(G)** Representative western blot results of the 5 biomarkers. **(H)** Relative protein expression levels of the 5 biomarkers (*n* = 3). *P < 0.05, **P < 0.01 vs the sham group.

As illustrated in Figures 4A,B, the deterioration of cardiac morphology and function was observed in the ICM group. The serum BNP levels were significantly increased in the ICM group (Figure 4C). The results of HE and Masson staining showed that compared to the sham group, disorganized myocardial tissue, disrupted cell structure with an enlarged or dissolved nucleus, and massive deposited collagen fibres were observed in the ICM group (Figures 4D,E). Moreover, cardiomyocyte hypertrophy was also observed in the ICM group (Figure 4F). Taken together, the results above showed that the rat ICM model was successfully established. To further verify the results of bioinformatics analysis, we detected the protein expression levels of the 5 potential biomarkers in the myocardial tissues. Compared with the sham group, the protein expression levels of SERPINA3, FCN3, and CD163 were lower in the ICM group and the opposite was true for PTN and SCUBE2 (Figures 4G,H). Altogether, their changing trends were consistent with the results of bioinformatics analysis.

### Establishment and evaluation of the nomogram model

**FIGURE 5:**
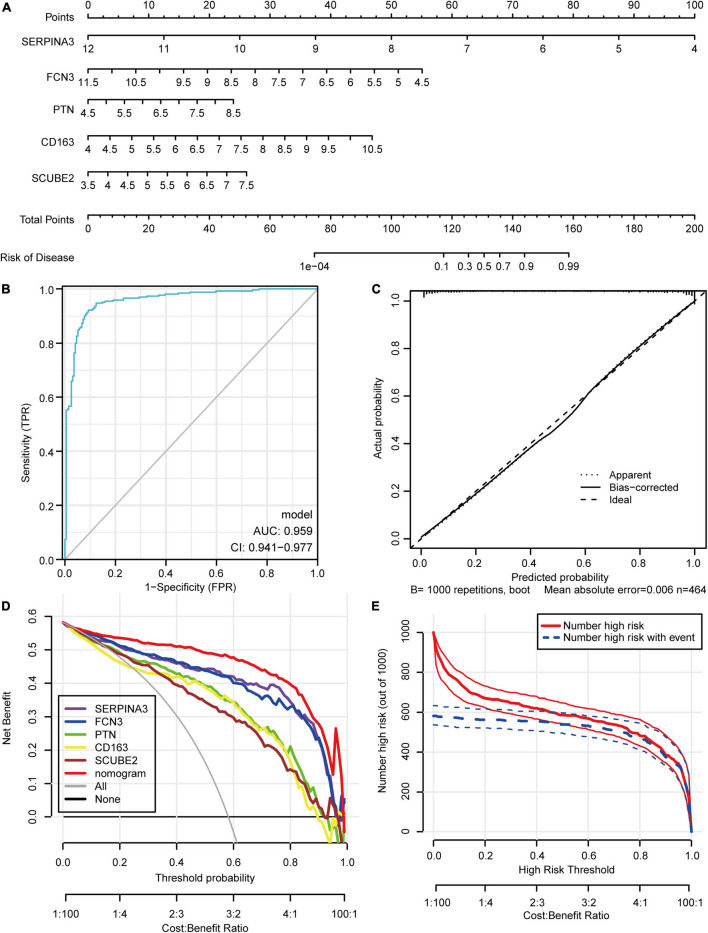
Construction and Evaluation of the nomogram model. **(A)** Construction of the nomogram model based on the 5 potential diagnostic biomarkers. **(B)** Receiver operating characteristic curve. **(C)** Calibration curve. **(D)** Decision curve analysis. **(E)** Clinical impact curve.

The nomogram model constructed based on the 5 potential diagnostic biomarkers was shown in Figure 5A. The ROC analysis yielded the AUC values of 0.959 (95% CI, 0.941–0.977), which suggested that the prediction efficiency of the nomogram model was good (Figure 5B). The calibration curve indicated brilliant agreement among the apparent curve, bias-corrected curve, and ideal curve (Figure 5C). DCA and CIC showed that patients could benefit from the nomogram at threshold probabilities of 0.01–0.98 (Figures 5D,E). The risk score of each individual in the merged dataset was obtained using the nomogram model (Supplementary Table 4).

### Functional enrichment analyses of the differentially expressed genes grouped by risk score

**FIGURE 6:**
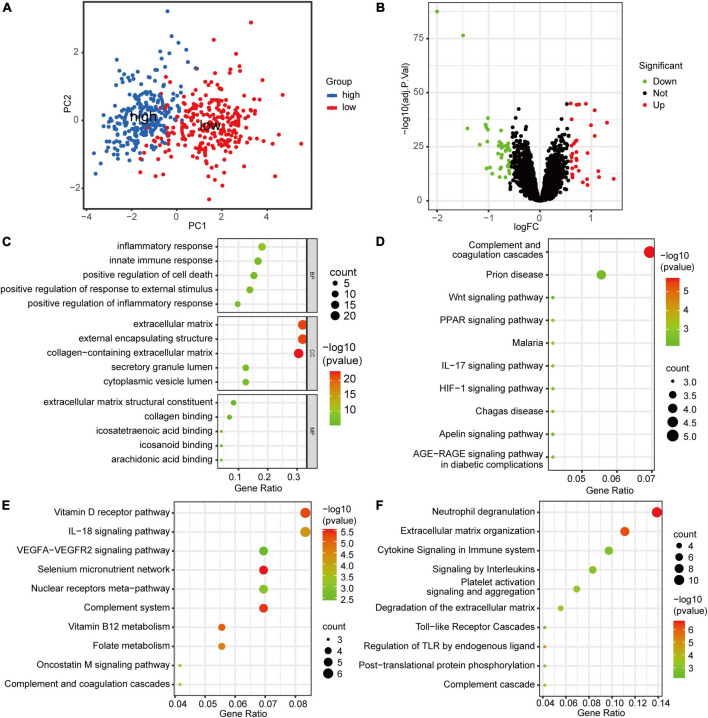
The Functional enrichment analyses of the DEGs grouped by risk score. **(A)** The principal component analysis cluster plot of low- and high-risk samples in the merged dataset. **(B)** Volcano plot of the DEGs between low- and high-risk groups. **(C)** The top five enriched GO terms for the DEGs between low- and high-risk groups in biological process, molecular function, and cellular component categories. **(D)** The ten KEGG pathways were closely related to ICM enriched by DEGs between the low- and high-risk groups. **(E)** The ten Wiki pathways were closely related to ICM enriched by DEGs between the low- and high-risk groups. **(F)** The ten Reactome pathways were closely related to ICM enriched by DEGs between the low- and high-risk groups.

The principal component analysis showed that the clusters of low- and high-risk groups significantly showed no overlap, which suggested that these 5 potential diagnostic biomarkers could be well differentiated for the presence or absence of ICM in individuals (Figure 6A). After screening with the threshold, 72 DEGs between the low- and high-risk groups were identified (Figure 6B). To facilitate the understanding of the functional mechanisms of 5 potential biomarkers regulating ICM, functional enrichment analyses were implemented. The results of the GO enrichment analysis showed 23 terms enriched for cellular components, 32 for molecular function, and 146 for biological processes. The GO terms closely associated with ICM were mainly collagen binding, extracellular matrix, inflammatory response, innate immune response, etc. (Figure 6C). Based on the pre-set screening criteria, we enriched 11, 40, and 17 pathways from the KEGG, Reactome, and Wiki databases, respectively. The enriched KEGG pathways closely associated with ICM were mainly the IL-17 signaling pathway, Apelin signaling pathway, PPAR signaling pathway, etc. (Figure 6D). The enriched Wiki pathways closely associated with ICM were mainly the VEGFA-VEGFR2 signaling pathway, IL-18 signaling pathway, complement, coagulation cascades, etc. (Figure 6E). The enriched Reactome pathways closely associated with ICM were mainly TLRs cascades, cytokine signaling in the immune system, neutrophil degranulation, etc. (Figure 6F).

## Discussion

Atherosclerosis, a chronic inflammatory disease of the arterial wall, is the common pathophysiological mechanism of many atherosclerotic diseases, such as coronary arterial disease, ischemic stroke, and ICM. There is ample evidence that inflammation has been identified as an important cardiovascular risk factor for atherosclerosis, which in turn leads to ICM (25). The accumulated evidence has highlighted the link between inflammatory burden and residual risk in ICM, which provides novel ideas and entry points in risk mitigation (25). The concept of management against residual inflammatory risk among patients with ICM is gaining increasing attention (25). Therefore, anti-inflammatory strategies represent attractive approaches for treating ICM (26). In sum, exploring the inflammatory mechanism associated with the occurrence and progression of ICM is of great significance for a deeper understanding of the pathological mechanism of ICM, early diagnosis, and identifying new therapeutic targets from the perspective of inflammation.

In our study, we combined 8 microarray datasets using the SVA package and identified 19 DEIRGs. Then, we annotated these DEIRGs and found that they were mainly involved in the inflammatory response, leukocyte chemotaxis and migration, response to bacterium and other processes, and enriched in the apelin signaling pathway, IL-17 and IL-18 signaling pathway, neutrophil degranulation, TLRs cascades, and other pathways. Neutrophils, the most plentiful kind of leukocytes, play an important role during key cellular and molecular events of ICM (27, 28). Neutrophil migration, chemotaxis, and degranulation are necessary for different pathological stages of ICM (27, 28). IL-17 and 18, as pro-inflammatory cytokines, are up-regulated in patients with ICM and related to the New York Heart Association (NYHA) class (29, 30). They play a pathogenic role in HF through several pathways including NF-κB, and MAPK signaling pathways (31, 32). TLRs, a family of pattern recognition receptors, play a pivotal role in immune responses and are closely associated with several cardiovascular diseases (33). In ICM, TLRs can induce activation of NF-κB and inflammatory responses, regulate cardiomyocyte apoptosis and alter cardiac function, with TLR2 and TLR4 playing the most important role (34). According to the above studies, the regulatory mechanisms of DEIRGs during ICM were related to inflammation-related biological processes and pathways, which might contribute to a better understanding of the pathologic mechanism of ICM.

Next, we used SVM-RFE, LASSO, and RF algorithms to identify 5 robust potential biomarkers (including SERPINA3, FCN3, PTN, CD163, and SCUBE2) from DEIRGs. The verified results of the functional experiment of the ICM rat showed that the protein expression levels of SERPINA3, FCN3, and CD163 were decreased in the ICM rat and the opposite was true for PTN and SCUBE2. Meanwhile, the validation results of RNA-Sequencing datasets showed that each of the 5 potential biomarkers showed good discriminant power, and their expression trends in RNA-Sequencing datasets were also consistent with the bioinformatics results. In addition, we established one risk prediction nomogram of ICM based on the 5 potential biomarkers and further tested its efficacy with moderate discrimination and good calibration. The results indicated that the nomogram may assist clinicians in effectively evaluating the risk and precise treatment of patients in ICM.

Next, the 5 potential biomarkers were analyzed and discussed for their association with ICM along with the literature. SERPINA3 belonging to the superfamily of serine protease inhibitors plays a significant role in the pathogenesis of atherosclerosis (35). SERPINA3 not only was a novel diagnostic and pharmacological target for HF but also associated with major adverse cardiovascular events in patients with acute myocardial infarction (36, 37). FCN3 is the most abundant and potent recognition molecule in the complement system lectin pathway and mediates autoimmune system and inflammation and other diseases (38). FCN3 can be used as a new biomarker for the prognosis of HF and has nothing to do with the etiology of HF (38, 39). In our study, SERPINA3 and FCN3 were down-regulated in bioinformatics analysis, the external validation data, and the functional experiment of the ICM rat. Jing et al. (40) found that SERPINA3 and FCN3 were identified as the potential biomarkers in ICM using bioinformatics analysis. The validation results of SERPINA3 and FCN3 in myocardial tissue of ICM patients were consistent with the results of our study.

PTN, a growth factor, plays an important role in nervous system development, tumor angiogenesis and growth, wound repair, and other disease phenotypes (41, 42). PTN may be a novel potential diagnostic and therapeutic target for dilated cardiomyopathy (36). Some studies have reported that PTN increased apoptosis of cardiomyocytes and enhanced neovasculature formation in ICM (42–44). SCUBE2, as a member of the SCUBE family, is expressed in a wide range of human tissues including cardiovascular tissues. SCUBE2 is involved in vascular endothelial function changes and vascular complications, particularly in diabetes and atherosclerosis (45). The results of our study suggested that SCUBE2 might be a new target that played an important role in ICM. CD163 mainly expressed in monocytes/macrophages is critical in inflammation. CD163 is involved in the progression of acute HF and also contributed to the pathogenesis of acute decompensated HF (46). Furthermore, it was reported that CD163-expressing macrophages not only regulated tissue regeneration after ischaemic injury induced by unilateral femoral artery ligation but also promoted ventricular functional recovery after myocardial infarction (47, 48). In sum, the unique findings of our study were that PTN, SCUBE2, and CD163 played an important role in the pathology of ICM and might be potential diagnostic and therapeutic targets for ICM. Meanwhile, we also innovatively integrated the 5 potential biomarkers (including SERPINA3, FCN3, PTN, CD163, and SCUBE2) for a multiple indicator co-diagnosis of ICM from an inflammatory perspective and constructed a nomogram that might facilitate accurate disease assessment of ICM patients by clinicians or researchers.

To facilitate an understanding of the functional mechanisms of 5 potential biomarkers regulating ICM, we next divided all samples into high and low-risk groups according to the nomogram model and carried out functional enrichment analyses of the DEGs between the two groups. The results revealed that these genes principally participated in the inflammatory response, cell death, and other processes, and enriched in the HIF-1 signaling pathway, neutrophil degranulation, TLRs cascades, signaling by interleukins, and other signaling pathways. The inflammatory response has been considered the major pathophysiological contributor to HF (49). Interleukins are a group of cytokines that are produced by and interact with a variety of cells and play an important role in the transmission of information, activation and regulation of immune cells, and inflammatory response. Interleukins, as major inflammatory mediators, have been reported to be closely associated with HF (49). In addition to IL-17 and 18 mentioned above, there are also IL-1, 6, 8, 10, and 33 (31). IL-1 cytokine family has several members, including IL-1, IL-33, ST2, IL-18, and others. The CANTOS study showed that canakinumab, a human monoclonal anti-human IL-1β antibody, could reduce the risk of major cardiovascular disease events in patients with a history of myocardial infarction without any effect on lipid levels, which demonstrated that anti-inflammatory therapy inhibiting the IL-1 pathway might indeed be beneficial for patients with coronary heart disease (50). During HF, IL-1 causes cardiac dysfunction and remodeling by stimulating cardiomyocyte apoptosis, favoring fibrosis, promoting arterial stiffness and microvascular inflammation, and other multiple mechanisms (49). Meanwhile, IL-1 also induces the activation of leukocytes and endothelial cells, which in turn promotes their interaction and increases the mobilization of inflammatory cells to the myocardium (49). The above studies suggest that the IL-1 pathway may be an important contributor to the progression of ICM and has emerged as an attractive target for the anti-inflammatory treatment of cardiovascular disease. The IL-33/ST2 signaling pathway mediated the inflammatory response in ICM patients (51). The IL-6 concentration was elevated in patients with ICM and proportional to the NYHA functional class (52, 53). Studies had shown that IL-6 mediated the pathological process of HF through JAK/STAT3, MAPKs, and PI3K/Akt pathway and other signaling pathways (32). In summary, the enriched pathway and process of the DEGs between high and low-risk groups were to some extent consistent with the pathological process of ICM shown in prior research. In the future, in-depth research should be done to verify the above findings.

Our study had the following strengths. First, a small sample size was an issue generally encountered in research for bioinformatics analysis, especially for non-oncological diseases. We attempted to mend the limitation by integrating multiple independent databases using the SVA package to guarantee that the results were convincing. Second, three ML algorithms were used to screen the potential inflammation-related biomarkers of ICM. Third, multiple RNA-Sequencing datasets and the functional experiment of the ICM rat were implemented to validate the obtained 5 potential inflammatory biomarkers, which could prevent the bias from the results of pure bioinformatics analysis. Finally, the inflammatory biomarker-based nomogram described in the study has not been reported previously and provides a new diagnostic predictive tool for ICM. However, there were several limitations in our research, which should be considered in drawing the conclusions. First, our findings are derived from currently available gene expression data from both microarray and RNA-seq technologies. Some genes, although playing potential roles in ICM, were overlooked in our study because they were not detected by existing assays. Second, the samples in the datasets used for our study were human myocardial tissue rather than peripheral blood. Therefore, it will be interesting to observe whether the potential inflammation-related diagnostic biomarkers mined with ML algorithms are amenable to measurement in blood samples. Third, the 5 potential inflammatory biomarkers had been validated by multiple independent datasets and an ICM animal model, which makes our results more convincing and accurate. However, it is important and urgent to validate these results using cardiac tissue from ICM patients. Finally, although internal validation was executed to test the validity of the nomogram, future studies are needed to externally validate the proposed nomograms. In addition, due to imperfect information in the included datasets, the clinical information in the nomogram was excluded. If clinical information closely related to ICM was added to the nomogram, this will improve the accurate screening and precise diagnosis of ICM.

## Conclusion

In conclusion, we have innovatively identified SERPINA3, FCN3, PTN, CD163, and SCUBE2 as potential biomarkers for ICM by combining bioinformatics analysis, ML algorithms, and the experimental verification strategy. Meanwhile, we also developed a nomogram model based on potential inflammation-related biomarkers to predict ICM. However, further studies should be performed in the future to verify the above findings. Our work may provide new and valuable insights into the mechanisms of ICM and its treatments from an inflammatory perspective.

## Data availability statement

The datasets presented in this study can be found in online repositories. The names of the repository/repositories and accession number(s) can be found in the article/Supplementary material.

## Ethics statement

The animal study was reviewed and approved by the animal experiment was approved by the Experimental Animal Welfare Ethics Review Committee of The First Affiliated Hospital of Henan University of Chinese Medicine (approval number, YFYDW2017005).

## Author contributions

MZ, JW, and JC: conceptualization. JW, XL, and JC: formal analysis and data curation. JW and SX: funding acquisition. SX, XL, YC, and JW: investigation. SX, JW, and YC: resources. XL and JW: visualization. JW and SX: writing – original draft. MZ and JC: writing – review and editing. All authors approved the final version of the manuscript.

## Footnotes

1
http://www.ncbi.nlm.nih.gov/geo

2
http://www.disgenet.org

3
http://www.gsea-msigdb.org/gsea/index.jsp

4
http://metascape.org
