# Supplementary material for: Identification of potential biomarkers of inflammation-related genes for ischemic cardiomyopathy
Source: Front Cardiovasc Med. 2022 Aug 23;9:972274. doi: 10.3389/fcvm.2022.972274 (PMC9445158; doi:10.3389/fcvm.2022.972274)
Supplement: Supplementary file 1 [file Data_Sheet_1.doc]

| 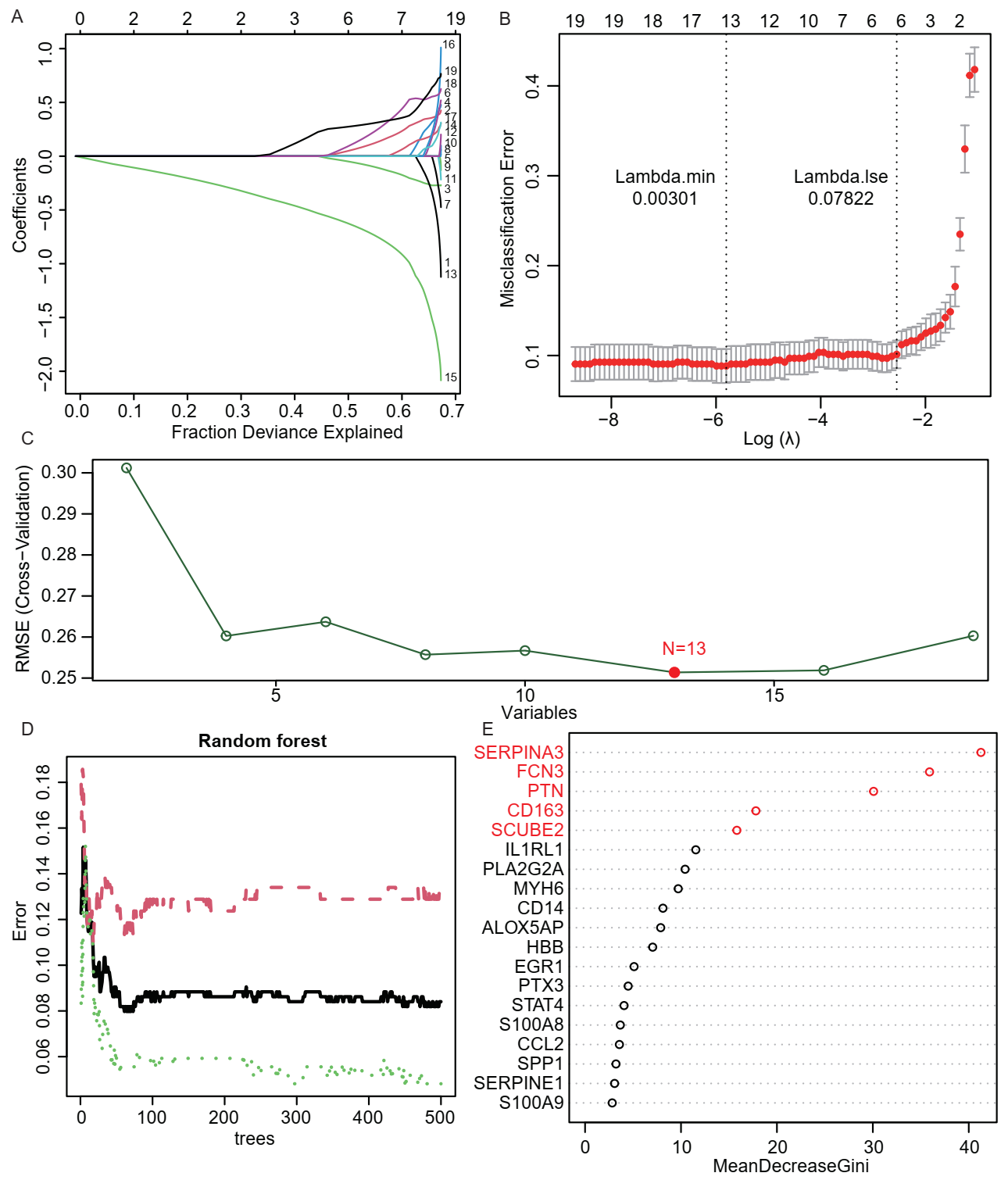 |
| --- |
| Supplementary Figure 1. Screening process of the biomarkers for ICM via three machine learning algorithms. (A) LASSO coefficient profiles of the 19 DEIRGs. (B) A plot of the biomarkers selection via LASSO. (C) A plot of the biomarkers selection via SVM-RFE. (D) Out-of-bag error rate curve for RF model. (E) The importance ranking of the DEIRGs based on the RF model. ICM: ischaemic cardiomyopathy. LASSO: least absolute shrinkage and selection operator. DEIRGs: differentially expressed inflammation-related genes. SVM-RFE: support vector machine recursive feature elimination. RF: random forest. |
