# Supplementary material for: Identification of potential biomarkers of inflammation-related genes for ischemic cardiomyopathy
Source: Front Cardiovasc Med. 2022 Aug 23;9:972274. doi: 10.3389/fcvm.2022.972274 (PMC9445158; doi:10.3389/fcvm.2022.972274)
Supplement: Supplementary file 2 [file Data_Sheet_2.doc]

| 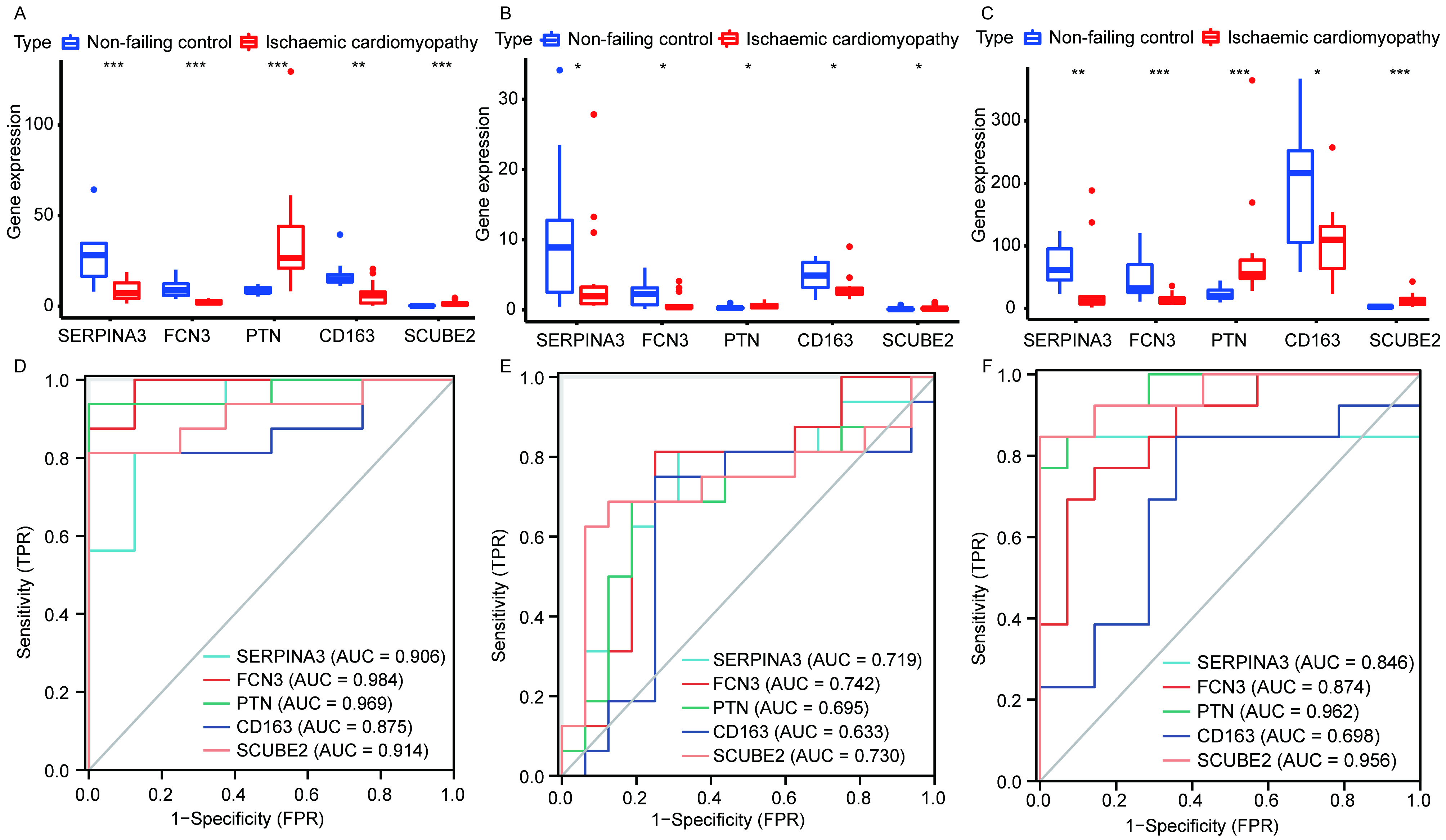 |
| --- |
| Supplementary figure 2. The results of external validation of the potential biomarkers in the RNA-Sequencing datasets. (A) The expression levels of the 5 potential biomarkers in the NFC and ICM samples from GSE46224 dataset. (B) The expression levels of 5 potential biomarkers in the NFC and ICM samples from GSE48166 dataset. (C) The expression levels of 5 potential biomarkers in the NFC and ICM samples from GSE116250 dataset. (D) ROC curve evaluation of the diagnostic effectiveness of the 5 potential biomarkers using GSE46224 dataset. Sky blue, SERPINA3, AUC: 0.906 (95% CI 0.780-1.000); Red, FCN3, AUC: 0.984 (95% CI 0.947-1.000); Green, PTN, AUC: 0.969 (95% CI 0.903-1.000);   Dark blue, CD163, AUC: 0.875 (95% CI 0.729-1.000);  Light red, SCUBE2, AUC: 0.914 (95% CI 0.800-1.000). (E) ROC curve evaluation of the diagnostic effectiveness of the 5 potential biomarkers using GSE48166 dataset. Sky blue, SERPINA3, AUC: 0.719 (95% CI 0.529-0.909); Red, FCN3, AUC: 0.742 (95% CI 0.558-0.926); Green, PTN, AUC: 0.695 (95% CI 0.497-0.893);   Dark blue, CD163, AUC: 0.633 (95% CI 0.417-0.848);  Light red, SCUBE2, AUC: 0.730 (95% CI 0.536-0.925). (F) ROC curve evaluation of the diagnostic effectiveness of the 5 potential biomarkers using GSE116250 dataset. Sky blue, SERPINA3, AUC: 0.846 (95% CI 0.642-1.000); Red, FCN3, AUC: 0.874 (95% CI 0.741-1.000); Green, PTN, AUC: 0.962 (95% CI 0.902-1.000);   Dark blue, CD163, AUC: 0.698 (95% CI 0.485-0.911);  Light red, SCUBE2, AUC: 0.956 (95% CI 0.883-1.000). |
