# Supplementary material for: Identification of potential biomarkers of inflammation-related genes for ischemic cardiomyopathy
Source: Front Cardiovasc Med. 2022 Aug 23;9:972274. doi: 10.3389/fcvm.2022.972274 (PMC9445158; doi:10.3389/fcvm.2022.972274)
Supplement: Supplementary file 3 [file Table_1.doc]

| Supplementary Table 1a The relevant information of selected datasets. | | | | |
| --- | --- | --- | --- | --- |
| Dataset ID | Platform | Sample size (NFC/ICM) | Tissues | Technology |
| GSE5406 | [GPL96](https://www.ncbi.nlm.nih.gov/geo/query/acc.cgi?acc=GPL96) | 16/108 | Left ventricular myocardium | Array |
| GSE16499 | [GPL5175](https://www.ncbi.nlm.nih.gov/geo/query/acc.cgi?acc=GPL5175) | 15/15 | Left ventricular myocardium | Array |
| GSE21610 | [GPL570](https://www.ncbi.nlm.nih.gov/geo/query/acc.cgi?acc=GPL570) | 8/18 | Left ventricular myocardium | Array |
| GSE42955 | [GPL6244](https://www.ncbi.nlm.nih.gov/geo/query/acc.cgi?acc=GPL6244) | 5/12 | Left ventricular myocardium | Array |
| GSE52601 | [GPL10558](https://www.ncbi.nlm.nih.gov/geo/query/acc.cgi?acc=GPL10558) | 4/8 | Left ventricular myocardium | Array |
| GSE1869 | [GPL96](https://www.ncbi.nlm.nih.gov/geo/query/acc.cgi?acc=GPL96) | 6/10 | Left ventricular myocardium | Array |
| GSE57338 | [GPL11532](https://www.ncbi.nlm.nih.gov/geo/query/acc.cgi?acc=GPL11532) | 136/95 | Left ventricular myocardium | Array |
| GSE76701 | [GPL570](https://www.ncbi.nlm.nih.gov/geo/query/acc.cgi?acc=GPL570) | 4/4 | Left ventricular myocardium | Array |
| GSE116250 | [GPL16791](https://www.ncbi.nlm.nih.gov/geo/query/acc.cgi?acc=GPL16791) | 14/13 | Left ventricular myocardium | RNA-seq |
| GSE48166 | [GPL9115](https://www.ncbi.nlm.nih.gov/geo/query/acc.cgi?acc=GPL9115) | 16/16 | Left ventricular myocardium | RNA-seq |
| GSE46224 | [GPL11154](https://www.ncbi.nlm.nih.gov/geo/query/acc.cgi?acc=GPL11154) | 8/16 | Left ventricular myocardium | RNA-seq |
| Abbreviation: NFC, non-failing control; ICM, ischemic cardiomyopathy. | | | | |

| Supplementary Table 1b The characteristics of population in the selected datasets. | | | | | | | |
| --- | --- | --- | --- | --- | --- | --- | --- |
| Dataset ID | Sample ID | Group | Age | Sex | Race | NYHA | EF(%) |
| GSE5406 | GSM123640 | NFC | / | / | / | / | / |
| GSE5406 | GSM123641 | NFC | / | / | / | / | / |
| GSE5406 | GSM123643 | NFC | / | / | / | / | / |
| GSE5406 | GSM123644 | NFC | / | / | / | / | / |
| GSE5406 | GSM123645 | NFC | / | / | / | / | / |
| GSE5406 | GSM123646 | NFC | / | / | / | / | / |
| GSE5406 | GSM123647 | NFC | / | / | / | / | / |
| GSE5406 | GSM123649 | NFC | / | / | / | / | / |
| GSE5406 | GSM123551 | NFC | / | / | / | / | / |
| GSE5406 | GSM123536 | NFC | / | / | / | / | / |
| GSE5406 | GSM123575 | NFC | / | / | / | / | / |
| GSE5406 | GSM123582 | NFC | / | / | / | / | / |
| GSE5406 | GSM123705 | NFC | / | / | / | / | / |
| GSE5406 | GSM123639 | NFC | / | / | / | / | / |
| GSE5406 | GSM123709 | NFC | / | / | / | / | / |
| GSE5406 | GSM123727 | NFC | / | / | / | / | / |
| GSE5406 | GSM123569 | ICM | / | / | / | / | / |
| GSE5406 | GSM123570 | ICM | / | / | / | / | / |
| GSE5406 | GSM123518 | ICM | / | / | / | / | / |
| GSE5406 | GSM123519 | ICM | / | / | / | / | / |
| GSE5406 | GSM123520 | ICM | / | / | / | / | / |
| GSE5406 | GSM123521 | ICM | / | / | / | / | / |
| GSE5406 | GSM123525 | ICM | / | / | / | / | / |
| GSE5406 | GSM123526 | ICM | / | / | / | / | / |
| GSE5406 | GSM123527 | ICM | / | / | / | / | / |
| GSE5406 | GSM123529 | ICM | / | / | / | / | / |
| GSE5406 | GSM123696 | ICM | / | / | / | / | / |
| GSE5406 | GSM123531 | ICM | / | / | / | / | / |
| GSE5406 | GSM123532 | ICM | / | / | / | / | / |
| GSE5406 | GSM123702 | ICM | / | / | / | / | / |
| GSE5406 | GSM123703 | ICM | / | / | / | / | / |
| GSE5406 | GSM123534 | ICM | / | / | / | / | / |
| GSE5406 | GSM123539 | ICM | / | / | / | / | / |
| GSE5406 | GSM123543 | ICM | / | / | / | / | / |
| GSE5406 | GSM123544 | ICM | / | / | / | / | / |
| GSE5406 | GSM123545 | ICM | / | / | / | / | / |
| GSE5406 | GSM123546 | ICM | / | / | / | / | / |
| GSE5406 | GSM123697 | ICM | / | / | / | / | / |
| GSE5406 | GSM123573 | ICM | / | / | / | / | / |
| GSE5406 | GSM123574 | ICM | / | / | / | / | / |
| GSE5406 | GSM123576 | ICM | / | / | / | / | / |
| GSE5406 | GSM123577 | ICM | / | / | / | / | / |
| GSE5406 | GSM123579 | ICM | / | / | / | / | / |
| GSE5406 | GSM123580 | ICM | / | / | / | / | / |
| GSE5406 | GSM123581 | ICM | / | / | / | / | / |
| GSE5406 | GSM123583 | ICM | / | / | / | / | / |
| GSE5406 | GSM123584 | ICM | / | / | / | / | / |
| GSE5406 | GSM123585 | ICM | / | / | / | / | / |
| GSE5406 | GSM123698 | ICM | / | / | / | / | / |
| GSE5406 | GSM123594 | ICM | / | / | / | / | / |
| GSE5406 | GSM123595 | ICM | / | / | / | / | / |
| GSE5406 | GSM123596 | ICM | / | / | / | / | / |
| GSE5406 | GSM123598 | ICM | / | / | / | / | / |
| GSE5406 | GSM123599 | ICM | / | / | / | / | / |
| GSE5406 | GSM123600 | ICM | / | / | / | / | / |
| GSE5406 | GSM123602 | ICM | / | / | / | / | / |
| GSE5406 | GSM123604 | ICM | / | / | / | / | / |
| GSE5406 | GSM123605 | ICM | / | / | / | / | / |
| GSE5406 | GSM123606 | ICM | / | / | / | / | / |
| GSE5406 | GSM123699 | ICM | / | / | / | / | / |
| GSE5406 | GSM123607 | ICM | / | / | / | / | / |
| GSE5406 | GSM123608 | ICM | / | / | / | / | / |
| GSE5406 | GSM123609 | ICM | / | / | / | / | / |
| GSE5406 | GSM123614 | ICM | / | / | / | / | / |
| GSE5406 | GSM123615 | ICM | / | / | / | / | / |
| GSE5406 | GSM123619 | ICM | / | / | / | / | / |
| GSE5406 | GSM123621 | ICM | / | / | / | / | / |
| GSE5406 | GSM123623 | ICM | / | / | / | / | / |
| GSE5406 | GSM123624 | ICM | / | / | / | / | / |
| GSE5406 | GSM123625 | ICM | / | / | / | / | / |
| GSE5406 | GSM123700 | ICM | / | / | / | / | / |
| GSE5406 | GSM123626 | ICM | / | / | / | / | / |
| GSE5406 | GSM123629 | ICM | / | / | / | / | / |
| GSE5406 | GSM123630 | ICM | / | / | / | / | / |
| GSE5406 | GSM123632 | ICM | / | / | / | / | / |
| GSE5406 | GSM123634 | ICM | / | / | / | / | / |
| GSE5406 | GSM123637 | ICM | / | / | / | / | / |
| GSE5406 | GSM123638 | ICM | / | / | / | / | / |
| GSE5406 | GSM123706 | ICM | / | / | / | / | / |
| GSE5406 | GSM123707 | ICM | / | / | / | / | / |
| GSE5406 | GSM123708 | ICM | / | / | / | / | / |
| GSE5406 | GSM123701 | ICM | / | / | / | / | / |
| GSE5406 | GSM123710 | ICM | / | / | / | / | / |
| GSE5406 | GSM123712 | ICM | / | / | / | / | / |
| GSE5406 | GSM123642 | ICM | / | / | / | / | / |
| GSE5406 | GSM123648 | ICM | / | / | / | / | / |
| GSE5406 | GSM123713 | ICM | / | / | / | / | / |
| GSE5406 | GSM123715 | ICM | / | / | / | / | / |
| GSE5406 | GSM123716 | ICM | / | / | / | / | / |
| GSE5406 | GSM123655 | ICM | / | / | / | / | / |
| GSE5406 | GSM123656 | ICM | / | / | / | / | / |
| GSE5406 | GSM123659 | ICM | / | / | / | / | / |
| GSE5406 | GSM123722 | ICM | / | / | / | / | / |
| GSE5406 | GSM123660 | ICM | / | / | / | / | / |
| GSE5406 | GSM123664 | ICM | / | / | / | / | / |
| GSE5406 | GSM123665 | ICM | / | / | / | / | / |
| GSE5406 | GSM123666 | ICM | / | / | / | / | / |
| GSE5406 | GSM123669 | ICM | / | / | / | / | / |
| GSE5406 | GSM123671 | ICM | / | / | / | / | / |
| GSE5406 | GSM123672 | ICM | / | / | / | / | / |
| GSE5406 | GSM123673 | ICM | / | / | / | / | / |
| GSE5406 | GSM123674 | ICM | / | / | / | / | / |
| GSE5406 | GSM123676 | ICM | / | / | / | / | / |
| GSE5406 | GSM123723 | ICM | / | / | / | / | / |
| GSE5406 | GSM123678 | ICM | / | / | / | / | / |
| GSE5406 | GSM123679 | ICM | / | / | / | / | / |
| GSE5406 | GSM123680 | ICM | / | / | / | / | / |
| GSE5406 | GSM123684 | ICM | / | / | / | / | / |
| GSE5406 | GSM123685 | ICM | / | / | / | / | / |
| GSE5406 | GSM123690 | ICM | / | / | / | / | / |
| GSE5406 | GSM123691 | ICM | / | / | / | / | / |
| GSE5406 | GSM123692 | ICM | / | / | / | / | / |
| GSE5406 | GSM123694 | ICM | / | / | / | / | / |
| GSE5406 | GSM123548 | ICM | / | / | / | / | / |
| GSE5406 | GSM123549 | ICM | / | / | / | / | / |
| GSE5406 | GSM123552 | ICM | / | / | / | / | / |
| GSE5406 | GSM123553 | ICM | / | / | / | / | / |
| GSE5406 | GSM123556 | ICM | / | / | / | / | / |
| GSE5406 | GSM123558 | ICM | / | / | / | / | / |
| GSE5406 | GSM123561 | ICM | / | / | / | / | / |
| GSE5406 | GSM123562 | ICM | / | / | / | / | / |
| GSE5406 | GSM123567 | ICM | / | / | / | / | / |
| GSE5406 | GSM123695 | ICM | / | / | / | / | / |
| GSE5406 | GSM123726 | ICM | / | / | / | / | / |
| GSE16499 | GSM414642 | NFC | / | / | / | / | / |
| GSE16499 | GSM414651 | NFC | / | / | / | / | / |
| GSE16499 | GSM414652 | NFC | / | / | / | / | / |
| GSE16499 | GSM414653 | NFC | / | / | / | / | / |
| GSE16499 | GSM414654 | NFC | / | / | / | / | / |
| GSE16499 | GSM414655 | NFC | / | / | / | / | / |
| GSE16499 | GSM414656 | NFC | / | / | / | / | / |
| GSE16499 | GSM414643 | NFC | / | / | / | / | / |
| GSE16499 | GSM414644 | NFC | / | / | / | / | / |
| GSE16499 | GSM414645 | NFC | / | / | / | / | / |
| GSE16499 | GSM414646 | NFC | / | / | / | / | / |
| GSE16499 | GSM414647 | NFC | / | / | / | / | / |
| GSE16499 | GSM414648 | NFC | / | / | / | / | / |
| GSE16499 | GSM414649 | NFC | / | / | / | / | / |
| GSE16499 | GSM414650 | NFC | / | / | / | / | / |
| GSE16499 | GSM414657 | ICM | / | / | / | / | / |
| GSE16499 | GSM414666 | ICM | / | / | / | / | / |
| GSE16499 | GSM414667 | ICM | / | / | / | / | / |
| GSE16499 | GSM414668 | ICM | / | / | / | / | / |
| GSE16499 | GSM414669 | ICM | / | / | / | / | / |
| GSE16499 | GSM414670 | ICM | / | / | / | / | / |
| GSE16499 | GSM414671 | ICM | / | / | / | / | / |
| GSE16499 | GSM414658 | ICM | / | / | / | / | / |
| GSE16499 | GSM414659 | ICM | / | / | / | / | / |
| GSE16499 | GSM414660 | ICM | / | / | / | / | / |
| GSE16499 | GSM414661 | ICM | / | / | / | / | / |
| GSE16499 | GSM414662 | ICM | / | / | / | / | / |
| GSE16499 | GSM414663 | ICM | / | / | / | / | / |
| GSE16499 | GSM414664 | ICM | / | / | / | / | / |
| GSE16499 | GSM414665 | ICM | / | / | / | / | / |
| GSE21610 | GSM545657 | NFC | 18 | Female | / | / | / |
| GSE21610 | GSM545658 | NFC | 40 | Male | / | / | / |
| GSE21610 | GSM545659 | NFC | 5 | Male | / | / | / |
| GSE21610 | GSM545660 | NFC | 28 | Male | / | / | / |
| GSE21610 | GSM545661 | NFC | 64 | Male | / | / | / |
| GSE21610 | GSM545662 | NFC | 31 | Male | / | / | / |
| GSE21610 | GSM545663 | NFC | 23 | Male | / | / | / |
| GSE21610 | GSM545664 | NFC | 23 | Female | / | / | / |
| GSE21610 | GSM545670 | ICM | 52 | Male | / | / | / |
| GSE21610 | GSM545672 | ICM | 66 | Male | / | / | / |
| GSE21610 | GSM545675 | ICM | 67 | Male | / | / | / |
| GSE21610 | GSM545676 | ICM | 52 | Male | / | / | / |
| GSE21610 | GSM545679 | ICM | 63 | Male | / | / | / |
| GSE21610 | GSM545680 | ICM | 43 | Male | / | / | / |
| GSE21610 | GSM545687 | ICM | 68 | Male | / | / | / |
| GSE21610 | GSM545691 | ICM | 57 | Male | / | / | / |
| GSE21610 | GSM545694 | ICM | 50 | Male | / | / | / |
| GSE21610 | GSM545700 | ICM | 52 | Male | / | / | / |
| GSE21610 | GSM545702 | ICM | 66 | Male | / | / | / |
| GSE21610 | GSM545705 | ICM | 67 | Male | / | / | / |
| GSE21610 | GSM545706 | ICM | 52 | Male | / | / | / |
| GSE21610 | GSM545709 | ICM | 63 | Male | / | / | / |
| GSE21610 | GSM545710 | ICM | 43 | Male | / | / | / |
| GSE21610 | GSM545717 | ICM | 68 | Male | / | / | / |
| GSE21610 | GSM545721 | ICM | 57 | Male | / | / | / |
| GSE21610 | GSM545724 | ICM | 50 | Male | / | / | / |
| GSE42955 | GSM1053922 | NFC | / | Male | / | / | / |
| GSE42955 | GSM1053929 | NFC | / | Male | / | / | / |
| GSE42955 | GSM1053939 | NFC | / | Male | / | / | / |
| GSE42955 | GSM1053940 | NFC | / | Male | / | / | / |
| GSE42955 | GSM1053942 | NFC | / | Male | / | / | / |
| GSE42955 | GSM1053914 | ICM | / | Male | / | / | / |
| GSE42955 | GSM1053920 | ICM | / | Male | / | / | / |
| GSE42955 | GSM1053921 | ICM | / | Male | / | / | / |
| GSE42955 | GSM1053923 | ICM | / | Male | / | / | / |
| GSE42955 | GSM1053927 | ICM | / | Male | / | / | / |
| GSE42955 | GSM1053928 | ICM | / | Male | / | / | / |
| GSE42955 | GSM1053930 | ICM | / | Male | / | / | / |
| GSE42955 | GSM1053931 | ICM | / | Male | / | / | / |
| GSE42955 | GSM1053916 | ICM | / | Male | / | / | / |
| GSE42955 | GSM1053932 | ICM | / | Male | / | / | / |
| GSE42955 | GSM1053934 | ICM | / | Male | / | / | / |
| GSE42955 | GSM1053936 | ICM | / | Male | / | / | / |
| GSE52601 | GSM1272369 | NFC | 60 | Male | Caucasian | / | 65 |
| GSE52601 | GSM1272378 | NFC | 56 | Male | Caucasian | / | 57 |
| GSE52601 | GSM1272382 | NFC | 40 | Male | Asian | / | / |
| GSE52601 | GSM1272388 | NFC | 2 | Male | Caucasian | / | / |
| GSE52601 | GSM1272372 | ICM | 68 | Male | Caucasian | 4 | 20 |
| GSE52601 | GSM1272374 | ICM | 68 | Male | Caucasian | 4 | 20 |
| GSE52601 | GSM1272376 | ICM | 64 | Male | Caucasian | 4 | 10 |
| GSE52601 | GSM1272379 | ICM | 64 | Male | Caucasian | 4 | 10 |
| GSE52601 | GSM1272380 | ICM | 70 | Female | Caucasian | 4 | 21 |
| GSE52601 | GSM1272386 | ICM | 70 | Female | Caucasian | 4 | 20 |
| GSE52601 | GSM1272387 | ICM | 66 | Male | Caucasian | 4 | 20 |
| GSE52601 | GSM1272391 | ICM | 66 | Male | Caucasian | 4 | 21 |
| GSE1869 | GSM33108 | NFC | / | / | / | / | / |
| GSE1869 | GSM33109 | NFC | / | / | / | / | / |
| GSE1869 | GSM33110 | NFC | / | / | / | / | / |
| GSE1869 | GSM33111 | NFC | / | / | / | / | / |
| GSE1869 | GSM33112 | NFC | / | / | / | / | / |
| GSE1869 | GSM33113 | NFC | / | / | / | / | / |
| GSE1869 | GSM33092 | ICM | / | / | / | / | / |
| GSE1869 | GSM33094 | ICM | / | / | / | / | / |
| GSE1869 | GSM33096 | ICM | / | / | / | / | / |
| GSE1869 | GSM33114 | ICM | / | / | / | / | / |
| GSE1869 | GSM33116 | ICM | / | / | / | / | / |
| GSE1869 | GSM33117 | ICM | / | / | / | / | / |
| GSE1869 | GSM33118 | ICM | / | / | / | / | / |
| GSE1869 | GSM33119 | ICM | / | / | / | / | / |
| GSE1869 | GSM33120 | ICM | / | / | / | / | / |
| GSE1869 | GSM33121 | ICM | / | / | / | / | / |
| GSE57338 | GSM1379830 | NFC | 52 | Female | / | / | / |
| GSE57338 | GSM1379831 | NFC | 80 | Female | / | / | / |
| GSE57338 | GSM1379832 | NFC | 55 | Female | / | / | / |
| GSE57338 | GSM1379833 | NFC | 61 | Male | / | / | / |
| GSE57338 | GSM1379834 | NFC | 24 | Female | / | / | / |
| GSE57338 | GSM1379835 | NFC | 57 | Female | / | / | / |
| GSE57338 | GSM1379836 | NFC | 59 | Female | / | / | / |
| GSE57338 | GSM1379837 | NFC | 56 | Male | / | / | / |
| GSE57338 | GSM1379838 | NFC | 67 | Female | / | / | / |
| GSE57338 | GSM1379839 | NFC | 56 | Male | / | / | / |
| GSE57338 | GSM1379840 | NFC | 42 | Male | / | / | / |
| GSE57338 | GSM1379841 | NFC | 58 | Male | / | / | / |
| GSE57338 | GSM1379842 | NFC | 60 | Female | / | / | / |
| GSE57338 | GSM1379843 | NFC | 56 | Male | / | / | / |
| GSE57338 | GSM1379844 | NFC | 48 | Male | / | / | / |
| GSE57338 | GSM1379845 | NFC | 40 | Male | / | / | / |
| GSE57338 | GSM1379846 | NFC | 1 | Male | / | / | / |
| GSE57338 | GSM1379847 | NFC | 26 | Female | / | / | / |
| GSE57338 | GSM1379848 | NFC | 76 | Male | / | / | / |
| GSE57338 | GSM1379849 | NFC | 59 | Female | / | / | / |
| GSE57338 | GSM1379850 | NFC | 42 | Male | / | / | / |
| GSE57338 | GSM1379851 | NFC | 58 | Male | / | / | / |
| GSE57338 | GSM1379852 | NFC | 51 | Female | / | / | / |
| GSE57338 | GSM1379853 | NFC | 65 | Female | / | / | / |
| GSE57338 | GSM1379854 | NFC | 34 | Male | / | / | / |
| GSE57338 | GSM1379855 | NFC | 62 | Female | / | / | / |
| GSE57338 | GSM1379856 | NFC | 63 | Male | / | / | / |
| GSE57338 | GSM1379857 | NFC | 38 | Male | / | / | / |
| GSE57338 | GSM1379858 | NFC | 12 | Male | / | / | / |
| GSE57338 | GSM1379859 | NFC | 42 | Male | / | / | / |
| GSE57338 | GSM1379860 | NFC | 38 | Male | / | / | / |
| GSE57338 | GSM1379861 | NFC | 38 | Male | / | / | / |
| GSE57338 | GSM1379862 | NFC | 25 | Male | / | / | / |
| GSE57338 | GSM1379863 | NFC | 56 | Female | / | / | / |
| GSE57338 | GSM1379864 | NFC | 44 | Male | / | / | / |
| GSE57338 | GSM1379865 | NFC | 43 | Male | / | / | / |
| GSE57338 | GSM1379866 | NFC | 60 | Female | / | / | / |
| GSE57338 | GSM1379867 | NFC | 8 | Female | / | / | / |
| GSE57338 | GSM1379868 | NFC | 62 | Male | / | / | / |
| GSE57338 | GSM1379869 | NFC | 45 | Female | / | / | / |
| GSE57338 | GSM1379870 | NFC | 66 | Male | / | / | / |
| GSE57338 | GSM1379871 | NFC | 65 | Female | / | / | / |
| GSE57338 | GSM1379872 | NFC | 59 | Male | / | / | / |
| GSE57338 | GSM1379873 | NFC | 56 | Male | / | / | / |
| GSE57338 | GSM1379874 | NFC | 28 | Male | / | / | / |
| GSE57338 | GSM1379875 | NFC | 61 | Female | / | / | / |
| GSE57338 | GSM1379876 | NFC | 65 | Female | / | / | / |
| GSE57338 | GSM1379877 | NFC | 26 | Female | / | / | / |
| GSE57338 | GSM1379878 | NFC | 21 | Male | / | / | / |
| GSE57338 | GSM1379880 | NFC | 36 | Male | / | / | / |
| GSE57338 | GSM1379881 | NFC | 30 | Male | / | / | / |
| GSE57338 | GSM1379882 | NFC | 34 | Male | / | / | / |
| GSE57338 | GSM1379883 | NFC | 40 | Male | / | / | / |
| GSE57338 | GSM1379884 | NFC | 33 | Male | / | / | / |
| GSE57338 | GSM1379885 | NFC | 60 | Male | / | / | / |
| GSE57338 | GSM1379886 | NFC | 35 | Female | / | / | / |
| GSE57338 | GSM1379887 | NFC | 32 | Male | / | / | / |
| GSE57338 | GSM1379888 | NFC | 57 | Male | / | / | / |
| GSE57338 | GSM1379889 | NFC | 18 | Male | / | / | / |
| GSE57338 | GSM1379890 | NFC | 20 | Male | / | / | / |
| GSE57338 | GSM1379891 | NFC | 45 | Male | / | / | / |
| GSE57338 | GSM1379892 | NFC | 57 | Female | / | / | / |
| GSE57338 | GSM1379893 | NFC | 59 | Female | / | / | / |
| GSE57338 | GSM1379894 | NFC | 52 | Female | / | / | / |
| GSE57338 | GSM1379895 | NFC | 51 | Female | / | / | / |
| GSE57338 | GSM1379896 | NFC | 18 | Female | / | / | / |
| GSE57338 | GSM1379897 | NFC | 48 | Female | / | / | / |
| GSE57338 | GSM1379898 | NFC | 54 | Female | / | / | / |
| GSE57338 | GSM1379899 | NFC | 69 | Male | / | / | / |
| GSE57338 | GSM1379900 | NFC | 46 | Female | / | / | / |
| GSE57338 | GSM1379901 | NFC | 63 | Male | / | / | / |
| GSE57338 | GSM1379902 | NFC | 53 | Female | / | / | / |
| GSE57338 | GSM1379903 | NFC | 50 | Male | / | / | / |
| GSE57338 | GSM1379904 | NFC | 58 | Male | / | / | / |
| GSE57338 | GSM1379905 | NFC | 66 | Female | / | / | / |
| GSE57338 | GSM1379906 | NFC | 55 | Female | / | / | / |
| GSE57338 | GSM1379907 | NFC | 61 | Female | / | / | / |
| GSE57338 | GSM1379908 | NFC | 54 | Female | / | / | / |
| GSE57338 | GSM1379909 | NFC | 53 | Female | / | / | / |
| GSE57338 | GSM1379910 | NFC | 57 | Male | / | / | / |
| GSE57338 | GSM1379911 | NFC | 54 | Female | / | / | / |
| GSE57338 | GSM1379912 | NFC | 43 | Male | / | / | / |
| GSE57338 | GSM1379913 | NFC | 43 | Female | / | / | / |
| GSE57338 | GSM1379914 | NFC | 52 | Male | / | / | / |
| GSE57338 | GSM1379915 | NFC | 56 | Female | / | / | / |
| GSE57338 | GSM1379918 | NFC | 55 | Male | / | / | / |
| GSE57338 | GSM1379919 | NFC | 52 | Male | / | / | / |
| GSE57338 | GSM1379920 | NFC | 47 | Male | / | / | / |
| GSE57338 | GSM1379921 | NFC | 44 | Female | / | / | / |
| GSE57338 | GSM1379922 | NFC | 51 | Female | / | / | / |
| GSE57338 | GSM1379923 | NFC | 52 | Male | / | / | / |
| GSE57338 | GSM1379924 | NFC | 55 | Male | / | / | / |
| GSE57338 | GSM1379925 | NFC | 50 | Male | / | / | / |
| GSE57338 | GSM1379926 | NFC | 36 | Female | / | / | / |
| GSE57338 | GSM1379927 | NFC | 34 | Female | / | / | / |
| GSE57338 | GSM1379961 | NFC | 59 | Male | / | / | / |
| GSE57338 | GSM1379962 | NFC | 33 | Female | / | / | / |
| GSE57338 | GSM1379965 | NFC | 71 | Male | / | / | / |
| GSE57338 | GSM1379966 | NFC | 24 | Female | / | / | / |
| GSE57338 | GSM1379967 | NFC | 54 | Female | / | / | / |
| GSE57338 | GSM1379968 | NFC | 47 | Male | / | / | / |
| GSE57338 | GSM1379969 | NFC | 57 | Female | / | / | / |
| GSE57338 | GSM1379970 | NFC | 52 | Male | / | / | / |
| GSE57338 | GSM1379971 | NFC | 42 | Male | / | / | / |
| GSE57338 | GSM1379972 | NFC | 62 | Female | / | / | / |
| GSE57338 | GSM1379973 | NFC | 49 | Female | / | / | / |
| GSE57338 | GSM1379974 | NFC | 44 | Female | / | / | / |
| GSE57338 | GSM1379975 | NFC | 60 | Male | / | / | / |
| GSE57338 | GSM1379976 | NFC | 51 | Female | / | / | / |
| GSE57338 | GSM1379977 | NFC | 44 | Female | / | / | / |
| GSE57338 | GSM1379978 | NFC | 64 | Male | / | / | / |
| GSE57338 | GSM1379979 | NFC | 42 | Male | / | / | / |
| GSE57338 | GSM1379980 | NFC | 47 | Male | / | / | / |
| GSE57338 | GSM1379981 | NFC | 71 | Female | / | / | / |
| GSE57338 | GSM1379982 | NFC | 46 | Female | / | / | / |
| GSE57338 | GSM1379983 | NFC | 56 | Female | / | / | / |
| GSE57338 | GSM1379984 | NFC | 78 | Female | / | / | / |
| GSE57338 | GSM1379985 | NFC | 50 | Female | / | / | / |
| GSE57338 | GSM1379986 | NFC | 50 | Male | / | / | / |
| GSE57338 | GSM1379987 | NFC | 60 | Male | / | / | / |
| GSE57338 | GSM1379988 | NFC | 46 | Male | / | / | / |
| GSE57338 | GSM1379989 | NFC | 29 | Male | / | / | / |
| GSE57338 | GSM1379990 | NFC | 74 | Male | / | / | / |
| GSE57338 | GSM1379991 | NFC | 56 | Male | / | / | / |
| GSE57338 | GSM1380008 | NFC | 57 | Female | / | / | / |
| GSE57338 | GSM1380010 | NFC | 58 | Female | / | / | / |
| GSE57338 | GSM1380011 | NFC | 65 | Male | / | / | / |
| GSE57338 | GSM1380012 | NFC | 79 | Male | / | / | / |
| GSE57338 | GSM1380013 | NFC | 66 | Female | / | / | / |
| GSE57338 | GSM1380014 | NFC | 62 | Female | / | / | / |
| GSE57338 | GSM1380015 | NFC | 45 | Female | / | / | / |
| GSE57338 | GSM1380021 | NFC | 75 | Male | / | / | / |
| GSE57338 | GSM1380027 | NFC | 33 | Male | / | / | / |
| GSE57338 | GSM1380122 | NFC | 56 | Male | / | / | / |
| GSE57338 | GSM1380123 | NFC | 21 | Female | / | / | / |
| GSE57338 | GSM1380124 | NFC | 24 | Male | / | / | / |
| GSE57338 | GSM1379813 | ICM | 62 | Female | / | / | / |
| GSE57338 | GSM1379814 | ICM | 62 | Male | / | / | / |
| GSE57338 | GSM1379815 | ICM | 51 | Male | / | / | / |
| GSE57338 | GSM1379819 | ICM | 55 | Female | / | / | / |
| GSE57338 | GSM1379821 | ICM | 66 | Male | / | / | / |
| GSE57338 | GSM1379822 | ICM | 67 | Male | / | / | / |
| GSE57338 | GSM1379823 | ICM | 67 | Male | / | / | / |
| GSE57338 | GSM1379824 | ICM | 53 | Male | / | / | / |
| GSE57338 | GSM1379825 | ICM | 63 | Female | / | / | / |
| GSE57338 | GSM1379826 | ICM | 60 | Male | / | / | / |
| GSE57338 | GSM1379828 | ICM | 63 | Male | / | / | / |
| GSE57338 | GSM1379829 | ICM | 62 | Male | / | / | / |
| GSE57338 | GSM1379928 | ICM | 57 | Male | / | / | / |
| GSE57338 | GSM1379930 | ICM | 70 | Male | / | / | / |
| GSE57338 | GSM1379933 | ICM | 68 | Male | / | / | / |
| GSE57338 | GSM1379938 | ICM | 58 | Male | / | / | / |
| GSE57338 | GSM1379943 | ICM | 64 | Male | / | / | / |
| GSE57338 | GSM1379945 | ICM | 71 | Male | / | / | / |
| GSE57338 | GSM1379946 | ICM | 56 | Female | / | / | / |
| GSE57338 | GSM1379948 | ICM | 53 | Male | / | / | / |
| GSE57338 | GSM1379949 | ICM | 60 | Female | / | / | / |
| GSE57338 | GSM1379951 | ICM | 64 | Male | / | / | / |
| GSE57338 | GSM1379952 | ICM | 50 | Male | / | / | / |
| GSE57338 | GSM1379953 | ICM | 67 | Male | / | / | / |
| GSE57338 | GSM1379958 | ICM | 46 | Male | / | / | / |
| GSE57338 | GSM1379959 | ICM | 65 | Male | / | / | / |
| GSE57338 | GSM1379960 | ICM | 62 | Male | / | / | / |
| GSE57338 | GSM1379964 | ICM | 58 | Male | / | / | / |
| GSE57338 | GSM1379992 | ICM | 54 | Male | / | / | / |
| GSE57338 | GSM1379997 | ICM | 67 | Male | / | / | / |
| GSE57338 | GSM1379998 | ICM | 49 | Male | / | / | / |
| GSE57338 | GSM1379999 | ICM | 58 | Male | / | / | / |
| GSE57338 | GSM1380000 | ICM | 64 | Male | / | / | / |
| GSE57338 | GSM1380001 | ICM | 51 | Male | / | / | / |
| GSE57338 | GSM1380002 | ICM | 67 | Male | / | / | / |
| GSE57338 | GSM1380004 | ICM | 54 | Male | / | / | / |
| GSE57338 | GSM1380005 | ICM | 60 | Male | / | / | / |
| GSE57338 | GSM1380007 | ICM | 64 | Male | / | / | / |
| GSE57338 | GSM1380018 | ICM | 48 | Male | / | / | / |
| GSE57338 | GSM1380019 | ICM | 65 | Male | / | / | / |
| GSE57338 | GSM1380024 | ICM | 61 | Male | / | / | / |
| GSE57338 | GSM1380030 | ICM | 58 | Male | / | / | / |
| GSE57338 | GSM1380040 | ICM | 61 | Male | / | / | / |
| GSE57338 | GSM1380041 | ICM | 54 | Male | / | / | / |
| GSE57338 | GSM1380042 | ICM | 65 | Female | / | / | / |
| GSE57338 | GSM1380043 | ICM | 60 | Male | / | / | / |
| GSE57338 | GSM1380044 | ICM | 66 | Male | / | / | / |
| GSE57338 | GSM1380045 | ICM | 59 | Male | / | / | / |
| GSE57338 | GSM1380047 | ICM | 62 | Male | / | / | / |
| GSE57338 | GSM1380049 | ICM | 64 | Male | / | / | / |
| GSE57338 | GSM1380051 | ICM | 62 | Male | / | / | / |
| GSE57338 | GSM1380052 | ICM | 67 | Male | / | / | / |
| GSE57338 | GSM1380054 | ICM | 52 | Male | / | / | / |
| GSE57338 | GSM1380055 | ICM | 42 | Female | / | / | / |
| GSE57338 | GSM1380057 | ICM | 58 | Male | / | / | / |
| GSE57338 | GSM1380060 | ICM | 56 | Male | / | / | / |
| GSE57338 | GSM1380061 | ICM | 65 | Male | / | / | / |
| GSE57338 | GSM1380063 | ICM | 50 | Male | / | / | / |
| GSE57338 | GSM1380064 | ICM | 60 | Male | / | / | / |
| GSE57338 | GSM1380065 | ICM | 53 | Male | / | / | / |
| GSE57338 | GSM1380066 | ICM | 59 | Male | / | / | / |
| GSE57338 | GSM1380067 | ICM | 60 | Male | / | / | / |
| GSE57338 | GSM1380069 | ICM | 56 | Male | / | / | / |
| GSE57338 | GSM1380070 | ICM | 61 | Female | / | / | / |
| GSE57338 | GSM1380071 | ICM | 60 | Male | / | / | / |
| GSE57338 | GSM1380074 | ICM | 64 | Female | / | / | / |
| GSE57338 | GSM1380076 | ICM | 64 | Male | / | / | / |
| GSE57338 | GSM1380079 | ICM | 60 | Male | / | / | / |
| GSE57338 | GSM1380081 | ICM | 41 | Female | / | / | / |
| GSE57338 | GSM1380086 | ICM | 65 | Male | / | / | / |
| GSE57338 | GSM1380087 | ICM | 68 | Male | / | / | / |
| GSE57338 | GSM1380089 | ICM | 55 | Male | / | / | / |
| GSE57338 | GSM1380090 | ICM | 58 | Male | / | / | / |
| GSE57338 | GSM1380091 | ICM | 62 | Male | / | / | / |
| GSE57338 | GSM1380092 | ICM | 68 | Female | / | / | / |
| GSE57338 | GSM1380093 | ICM | 68 | Male | / | / | / |
| GSE57338 | GSM1380097 | ICM | 58 | Male | / | / | / |
| GSE57338 | GSM1380100 | ICM | 62 | Male | / | / | / |
| GSE57338 | GSM1380101 | ICM | 59 | Female | / | / | / |
| GSE57338 | GSM1380104 | ICM | 52 | Male | / | / | / |
| GSE57338 | GSM1380105 | ICM | 58 | Male | / | / | / |
| GSE57338 | GSM1380106 | ICM | 55 | Female | / | / | / |
| GSE57338 | GSM1380107 | ICM | 45 | Female | / | / | / |
| GSE57338 | GSM1380108 | ICM | 58 | Male | / | / | / |
| GSE57338 | GSM1380109 | ICM | 62 | Male | / | / | / |
| GSE57338 | GSM1380110 | ICM | 62 | Male | / | / | / |
| GSE57338 | GSM1380111 | ICM | 68 | Male | / | / | / |
| GSE57338 | GSM1380112 | ICM | 27 | Male | / | / | / |
| GSE57338 | GSM1380113 | ICM | 67 | Male | / | / | / |
| GSE57338 | GSM1380114 | ICM | 54 | Male | / | / | / |
| GSE57338 | GSM1380115 | ICM | 64 | Male | / | / | / |
| GSE57338 | GSM1380116 | ICM | 63 | Male | / | / | / |
| GSE57338 | GSM1380117 | ICM | 63 | Male | / | / | / |
| GSE57338 | GSM1380119 | ICM | 38 | Male | / | / | / |
| GSE57338 | GSM1380120 | ICM | 55 | Male | / | / | / |
| GSE76701 | GSM2035924 | NFC | / | / | / | / | / |
| GSE76701 | GSM2035925 | NFC | / | / | / | / | / |
| GSE76701 | GSM2035926 | NFC | / | / | / | / | / |
| GSE76701 | GSM2035927 | NFC | / | / | / | / | / |
| GSE76701 | GSM2035928 | ICM | / | / | / | / | / |
| GSE76701 | GSM2035929 | ICM | / | / | / | / | / |
| GSE76701 | GSM2035930 | ICM | / | / | / | / | / |
| GSE76701 | GSM2035931 | ICM | / | / | / | / | / |
| GSE116250 | GSM3219571 | NFC | 46 | Male | / | / | / |
| GSE116250 | GSM3219570 | NFC | 31 | Male | / | / | / |
| GSE116250 | GSM3219569 | NFC | 54 | Male | / | / | / |
| GSE116250 | GSM3219568 | NFC | 40 | Male | / | / | / |
| GSE116250 | GSM3219567 | NFC | 46 | Male | / | / | / |
| GSE116250 | GSM3219566 | NFC | 60 | Male | / | / | / |
| GSE116250 | GSM3219565 | NFC | 46 | Male | / | / | / |
| GSE116250 | GSM3219564 | NFC | 52 | Male | / | / | / |
| GSE116250 | GSM3219563 | NFC | 60 | Male | / | / | / |
| GSE116250 | GSM3219562 | NFC | 58 | Female | / | / | / |
| GSE116250 | GSM3219561 | NFC | 56 | Female | / | / | / |
| GSE116250 | GSM3219560 | NFC | 41 | Female | / | / | / |
| GSE116250 | GSM3219559 | NFC | 54 | Male | / | / | / |
| GSE116250 | GSM3219558 | NFC | 43 | Male | / | / | / |
| GSE116250 | GSM3219621 | ICM | 58 | Male | / | / | / |
| GSE116250 | GSM3219620 | ICM | 55 | Female | / | / | / |
| GSE116250 | GSM3219619 | ICM | 61 | Male | / | / | / |
| GSE116250 | GSM3219618 | ICM | 49 | Male | / | / | / |
| GSE116250 | GSM3219617 | ICM | 62 | Male | / | / | / |
| GSE116250 | GSM3219616 | ICM | 56 | Male | / | / | / |
| GSE116250 | GSM3219615 | ICM | 60 | Female | / | / | / |
| GSE116250 | GSM3219614 | ICM | 56 | Female | / | / | / |
| GSE116250 | GSM3219613 | ICM | 54 | Male | / | / | / |
| GSE116250 | GSM3219612 | ICM | 49 | Male | / | / | / |
| GSE116250 | GSM3219611 | ICM | 55 | Male | / | / | / |
| GSE116250 | GSM3219610 | ICM | 56 | Male | / | / | / |
| GSE116250 | GSM3219609 | ICM | 63 | Male | / | / | / |
| GSE48166 | GSM1171549 | NFC | / | / | / | / | / |
| GSE48166 | GSM1171550 | NFC | / | / | / | / | / |
| GSE48166 | GSM1171551 | NFC | / | / | / | / | / |
| GSE48166 | GSM1171552 | NFC | / | / | / | / | / |
| GSE48166 | GSM1171553 | NFC | / | / | / | / | / |
| GSE48166 | GSM1171554 | NFC | / | / | / | / | / |
| GSE48166 | GSM1171555 | NFC | / | / | / | / | / |
| GSE48166 | GSM1171556 | NFC | / | / | / | / | / |
| GSE48166 | GSM1171557 | NFC | / | / | / | / | / |
| GSE48166 | GSM1171558 | NFC | / | / | / | / | / |
| GSE48166 | GSM1171559 | NFC | / | / | / | / | / |
| GSE48166 | GSM1171560 | NFC | / | / | / | / | / |
| GSE48166 | GSM1171561 | NFC | / | / | / | / | / |
| GSE48166 | GSM1171562 | NFC | / | / | / | / | / |
| GSE48166 | GSM1171563 | NFC | / | / | / | / | / |
| GSE48166 | GSM1171564 | NFC | / | / | / | / | / |
| GSE48166 | GSM1171565 | ICM | / | / | / | / | / |
| GSE48166 | GSM1171566 | ICM | / | / | / | / | / |
| GSE48166 | GSM1171567 | ICM | / | / | / | / | / |
| GSE48166 | GSM1171568 | ICM | / | / | / | / | / |
| GSE48166 | GSM1171569 | ICM | / | / | / | / | / |
| GSE48166 | GSM1171570 | ICM | / | / | / | / | / |
| GSE48166 | GSM1171571 | ICM | / | / | / | / | / |
| GSE48166 | GSM1171572 | ICM | / | / | / | / | / |
| GSE48166 | GSM1171573 | ICM | / | / | / | / | / |
| GSE48166 | GSM1171574 | ICM | / | / | / | / | / |
| GSE48166 | GSM1171575 | ICM | / | / | / | / | / |
| GSE48166 | GSM1171576 | ICM | / | / | / | / | / |
| GSE48166 | GSM1171577 | ICM | / | / | / | / | / |
| GSE48166 | GSM1171578 | ICM | / | / | / | / | / |
| GSE48166 | GSM1171579 | ICM | / | / | / | / | / |
| GSE48166 | GSM1171580 | ICM | / | / | / | / | / |
| GSE46224 | GSM1126612 | NFC | / | / | / | / | / |
| GSE46224 | GSM1126613 | NFC | / | / | / | / | / |
| GSE46224 | GSM1126614 | NFC | / | / | / | / | / |
| GSE46224 | GSM1126615 | NFC | / | / | / | / | / |
| GSE46224 | GSM1126616 | NFC | / | / | / | / | / |
| GSE46224 | GSM1126617 | NFC | / | / | / | / | / |
| GSE46224 | GSM1126618 | NFC | / | / | / | / | / |
| GSE46224 | GSM1126619 | NFC | / | / | / | / | / |
| GSE46224 | GSM1126620 | ICM | / | / | / | / | / |
| GSE46224 | GSM1126621 | ICM | / | / | / | / | / |
| GSE46224 | GSM1126622 | ICM | / | / | / | / | / |
| GSE46224 | GSM1126623 | ICM | / | / | / | / | / |
| GSE46224 | GSM1126624 | ICM | / | / | / | / | / |
| GSE46224 | GSM1126625 | ICM | / | / | / | / | / |
| GSE46224 | GSM1126626 | ICM | / | / | / | / | / |
| GSE46224 | GSM1126627 | ICM | / | / | / | / | / |
| GSE46224 | GSM1126636 | ICM | / | / | / | / | / |
| GSE46224 | GSM1126637 | ICM | / | / | / | / | / |
| GSE46224 | GSM1126638 | ICM | / | / | / | / | / |
| GSE46224 | GSM1126639 | ICM | / | / | / | / | / |
| GSE46224 | GSM1126640 | ICM | / | / | / | / | / |
| GSE46224 | GSM1126641 | ICM | / | / | / | / | / |
| GSE46224 | GSM1126642 | ICM | / | / | / | / | / |
| GSE46224 | GSM1126643 | ICM | / | / | / | / | / |

Abbreviation: NFC, non-failing control; ICM, ischemic cardiomyopathy; NYHA, New York Heart Association; EF, ejection fraction; "/" represents missing information.
