# Supplementary material for: Identification of potential biomarkers of inflammation-related genes for ischemic cardiomyopathy
Source: Front Cardiovasc Med. 2022 Aug 23;9:972274. doi: 10.3389/fcvm.2022.972274 (PMC9445158; doi:10.3389/fcvm.2022.972274)
Supplement: Supplementary file 4 [file Table_2.doc]

Supplementary Table 2. The results of significant GO enrichment analysis of DEIRGs.

| Ontology | GO ID | Description | Adj. P-value | Genes in the ontology |
| --- | --- | --- | --- | --- |
| BP | GO:0006954 | inflammatory response | 2.58145E-11 | SERPINA3,ALOX5AP,CD14,PLA2G2A,PTN,PTX3,S100A8,S100A9,CCL2,SPP1,CD163 |
| BP | GO:0050729 | positive regulation of inflammatory response | 2.18727E-06 | ALOX5AP,SERPINE1,PLA2G2A,S100A8,S100A9,IL1RL1,PTN,CD14,CCL2,PTX3,EGR1,HBB,SERPINA3,MYH6,SPP1 |
| BP | GO:0032103 | positive regulation of response to external stimulus | 3.04351E-05 | ALOX5AP,SERPINE1,PLA2G2A,PTN,S100A8,S100A9,IL1RL1 |
| BP | GO:0031349 | positive regulation of defense response | 6.85005E-05 | ALOX5AP,SERPINE1,PLA2G2A,S100A8,S100A9,IL1RL1 |
| BP | GO:0050727 | regulation of inflammatory response | 0.000343165 | ALOX5AP,SERPINE1,PLA2G2A,S100A8,S100A9,IL1RL1 |
| BP | GO:0002523 | leukocyte migration involved in inflammatory response | 0.000521513 | PTN,S100A8,S100A9 |
| BP | GO:0032496 | response to lipopolysaccharide | 0.002839982 | CD14,SERPINE1,S100A8,S100A9,CCL2 |
| BP | GO:0002237 | response to molecule of bacterial origin | 0.003135652 | CD14,SERPINE1,S100A8,S100A9,CCL2 |
| BP | GO:0030595 | leukocyte chemotaxis | 0.003135652 | PTN,S100A8,S100A9,CCL2 |
| BP | GO:0031347 | regulation of defense response | 0.003135652 | ALOX5AP,SERPINE1,PLA2G2A,S100A8,S100A9,IL1RL1 |
| BP | GO:0009617 | response to bacterium | 0.005489223 | CD14,SERPINE1,PLA2G2A,S100A8,S100A9,CCL2 |
| BP | GO:0045087 | innate immune response | 0.006744136 | CD14,PTX3,S100A8,S100A9,CCL2,FCN3,SERPINE1 |
| BP | GO:0009620 | response to fungus | 0.007947871 | PTX3,S100A8,S100A9 |
| BP | GO:0060326 | cell chemotaxis | 0.008426855 | PTN,S100A8,S100A9,CCL2 |
| BP | GO:0042698 | ovulation cycle | 0.010118117 | EGR1,PTN,PTX3,SERPINE1,SPP1,S100A9 |
| BP | GO:0050900 | leukocyte migration | 0.010875826 | PTN,S100A8,S100A9,CCL2 |
| BP | GO:0050766 | positive regulation of phagocytosis | 0.010875826 | PTX3,CCL2,FCN3 |
| BP | GO:0002526 | acute inflammatory response | 0.011847365 | SERPINA3,S100A8,CD163 |
| BP | GO:0060627 | regulation of vesicle-mediated transport | 0.012434771 | CD14,SERPINE1,PTX3,CCL2,FCN3 |
| BP | GO:0030593 | neutrophil chemotaxis | 0.01246725 | S100A8,S100A9,CCL2 |
| BP | GO:0071621 | granulocyte chemotaxis | 0.01427833 | S100A8,S100A9,CCL2 |
| BP | GO:1990266 | neutrophil migration | 0.016035315 | S100A8,S100A9,CCL2 |
| BP | GO:0098869 | cellular oxidant detoxification | 0.016035315 | ALOX5AP,HBB,S100A9,CD14,S100A8 |
| BP | GO:0010942 | positive regulation of cell death | 0.018463279 | EGR1,HBB,S100A8,S100A9,CCL2 |
| BP | GO:0097530 | granulocyte migration | 0.018463279 | S100A8,S100A9,CCL2 |
| BP | GO:0050764 | regulation of phagocytosis | 0.018871083 | PTX3,CCL2,FCN3 |
| BP | GO:1990748 | cellular detoxification | 0.021666959 | ALOX5AP,HBB,S100A9 |
| BP | GO:0097237 | cellular response to toxic substance | 0.026012546 | ALOX5AP,HBB,S100A9 |
| BP | GO:0042742 | defense response to bacterium | 0.0307417 | SERPINE1,PLA2G2A,S100A8,S100A9 |
| BP | GO:0019221 | cytokine-mediated signaling pathway | 0.030984773 | EGR1,CCL2,STAT4,IL1RL1,CD14,SPP1,PLA2G2A |
| BP | GO:0002688 | regulation of leukocyte chemotaxis | 0.030984773 | SERPINE1,PTN,CCL2,CD14,EGR1,IL1RL1,SPP1 |
| BP | GO:0097529 | myeloid leukocyte migration | 0.032228496 | S100A8,S100A9,CCL2 |
| BP | GO:0098754 | detoxification | 0.03505239 | ALOX5AP,HBB,S100A9 |
| BP | GO:0034097 | response to cytokine | 0.049470795 | CD14,EGR1,CCL2,STAT4,IL1RL1 |
| CC | GO:0031012 | extracellular matrix | 0.000572477 | SERPINA3,SERPINE1,PTX3,S100A8,S100A9,FCN3 |
| CC | GO:0030312 | external encapsulating structure | 0.000572477 | SERPINA3,SERPINE1,PTX3,S100A8,S100A9,FCN3 |
| CC | GO:0034774 | secretory granule lumen | 0.000572477 | SERPINA3,SERPINE1,PTX3,S100A8,S100A9 |
| CC | GO:0060205 | cytoplasmic vesicle lumen | 0.000572477 | SERPINA3,SERPINE1,PTX3,S100A8,S100A9 |
| CC | GO:0031983 | vesicle lumen | 0.000572477 | SERPINA3,SERPINE1,PTX3,S100A8,S100A9 |
| CC | GO:0062023 | collagen-containing extracellular matrix | 0.001734932 | SERPINA3,SERPINE1,S100A8,S100A9,FCN3 |
| CC | GO:0072562 | blood microparticle | 0.028552561 | SERPINA3,HBB,FCN3 |
| MF | GO:0050543 | icosatetraenoic acid binding | 1.01794E-05 | ALOX5AP,S100A8,S100A9 |
| MF | GO:0050544 | arachidonic acid binding | 1.01794E-05 | ALOX5AP,S100A8,S100A9 |
| MF | GO:0050542 | icosanoid binding | 1.18712E-05 | ALOX5AP,S100A8,S100A9 |
| MF | GO:0036041 | long-chain fatty acid binding | 0.000115377 | ALOX5AP,S100A8,S100A9 |
| MF | GO:0043177 | organic acid binding | 0.001613017 | ALOX5AP,HBB,S100A8,S100A9 |
| MF | GO:0005504 | fatty acid binding | 0.003072803 | ALOX5AP,S100A8,S100A9 |
| MF | GO:0008289 | lipid binding | 0.004553266 | ALOX5AP,CD14,PLA2G2A,S100A8,S100A9,SCUBE2 |
| MF | GO:0033293 | monocarboxylic acid binding | 0.010537588 | ALOX5AP,S100A8,S100A9 |
| MF | GO:0016209 | antioxidant activity | 0.010826116 | ALOX5AP,HBB,S100A9 |
| Note: BP: biological processes. CC: cellular component. MF: molecular function. | | | | |
