# Supplementary material for: Identification of potential biomarkers of inflammation-related genes for ischemic cardiomyopathy
Source: Front Cardiovasc Med. 2022 Aug 23;9:972274. doi: 10.3389/fcvm.2022.972274 (PMC9445158; doi:10.3389/fcvm.2022.972274)
Supplement: Supplementary file 5 [file Table_3.doc]

Supplementary Table 3. The results of significant pathways enrichment analysis of DEIRGs.

| Category | Term | Pathway Name | Adj. P-value | Genes in the pathway |
| --- | --- | --- | --- | --- |
| KEGG | hsa04657 | IL-17 signaling pathway | 0.006 | S100A8,S100A9,CCL2 |
| KEGG | hsa04933 | AGE-RAGE signaling pathway in diabetic complications | 0.006 | EGR1,SERPINE1,CCL2,SPP1 |
| KEGG | hsa04371 | Apelin signaling pathway | 0.010 | EGR1,SERPINE1,SPP1 |
| Reactome | R-HSA-5602498 | MyD88 deficiency (TLR2/4) | 0.000212873 | CD14,S100A8,S100A9 |
| Reactome | R-HSA-5603041 | IRAK4 deficiency (TLR2/4) | 0.000212873 | CD14,S100A8,S100A9 |
| Reactome | R-HSA-5686938 | Regulation of TLR by endogenous ligand | 0.000220881 | CD14,S100A8,S100A9 |
| Reactome | R-HSA-6798695 | Neutrophil degranulation | 0.000220881 | SERPINA3,CD14,HBB,PTX3,S100A8,S100A9 |
| Reactome | R-HSA-5260271 | Diseases of Immune System | 0.000388861 | CD14,S100A8,S100A9 |
| Reactome | R-HSA-5602358 | Diseases associated with the TLR signaling cascade | 0.000388861 | CD14,S100A8,S100A9 |
| Reactome | R-HSA-1236974 | ER-Phagosome pathway | 0.007267959 | CD14,S100A8,S100A9 |
| Reactome | R-HSA-6803157 | Antimicrobial peptides | 0.007267959 | PLA2G2A,S100A8,S100A9 |
| Reactome | R-HSA-166058 | MyD88:MAL(TIRAP) cascade initiated on plasma membrane | 0.007267959 | CD14,S100A8,S100A9 |
| Reactome | R-HSA-168188 | Toll Like Receptor TLR6:TLR2 Cascade | 0.007267959 | CD14,S100A8,S100A9 |
| Reactome | R-HSA-168179 | Toll Like Receptor TLR1:TLR2 Cascade | 0.007267959 | CD14,S100A8,S100A9 |
| Reactome | R-HSA-181438 | Toll Like Receptor 2 (TLR2) Cascade | 0.007267959 | CD14,S100A8,S100A9 |
| Reactome | R-HSA-1236975 | Antigen processing-Cross presentation | 0.007267959 | CD14,S100A8,S100A9 |
| Reactome | R-HSA-166016 | Toll Like Receptor 4 (TLR4) Cascade | 0.014265323 | CD14,S100A8,S100A9 |
| Reactome | R-HSA-168898 | Toll-like Receptor Cascades | 0.020825146 | CD14,S100A8,S100A9 |
| Wiki | WP15 | Selenium micronutrient network | 1.26104E-06 | SERPINA3,ALOX5AP,HBB,SERPINE1,CCL2,CD14,EGR1,CD163 |
| Wiki | WP5115 | Network map of SARS-CoV-2 signaling pathway | 1.26104E-06 | CD14,EGR1,HBB,SERPINE1,CCL2,CD163 |
| Wiki | WP1533 | Vitamin B12 metabolism | 7.51632E-06 | SERPINA3,HBB,SERPINE1,CCL2 |
| Wiki | WP176 | Folate metabolism | 1.66301E-05 | SERPINA3,HBB,SERPINE1,CCL2 |
| Wiki | WP2877 | Vitamin D receptor pathway | 1.59709E-05 | CD14,S100A8,S100A9,SPP1,IL1RL1 |
| Wiki | WP3624 | Lung fibrosis | 0.000945138 | PTX3,CCL2,SPP1,MYH6 |
| Wiki | WP4754 | IL-18 signaling pathway | 0.002173642 | MYH6,PTX3,CCL2,SPP1 |
| Wiki | WP2374 | Oncostatin M signaling pathway | 0.000945138 | EGR1,SERPINE1,CCL2,PLA2G2A,ALOX5AP,SCUBE2 |
| Wiki | WP2431 | Spinal cord injury | 0.004415763 | EGR1,PLA2G2A,CCL2 |
