# Supplementary material for: Identification of potential biomarkers of inflammation-related genes for ischemic cardiomyopathy
Source: Front Cardiovasc Med. 2022 Aug 23;9:972274. doi: 10.3389/fcvm.2022.972274 (PMC9445158; doi:10.3389/fcvm.2022.972274)
Supplement: Supplementary file 6 [file Table_4.doc]

Supplementary Table 4 The risk score of each individual in the merged dataset.

| IDStatus | SERPINA3 | FCN3 | PTN | CD163 | SCUBE2 | RiskScore | Risk |  |
| --- | --- | --- | --- | --- | --- | --- | --- | --- |
| GSE5406_GSM123551_con | 0 | 6.736489 | 7.696194 | 6.51988 | 7.013095 | 4.933873 | 0.797248687 | high |
| GSE16499_GSM414650_con | 0 | 7.496033 | 7.139416 | 6.053827 | 7.140338 | 4.943327 | 0.556958851 | low |
| GSE16499_GSM414656_con | 0 | 6.753972 | 8.190645 | 7.524969 | 6.470074 | 5.07527 | 0.767071499 | low |
| GSE57338_GSM1379835_con | 0 | 7.440064 | 7.964347 | 5.996843 | 7.938846 | 4.845338 | 0.515299719 | low |
| GSE57338_GSM1379842_con | 0 | 5.948596 | 7.856726 | 6.528954 | 7.572203 | 4.616839 | 0.957462361 | high |
| GSE57338_GSM1379845_con | 0 | 7.105339 | 8.608392 | 6.494687 | 7.180916 | 5.557434 | 0.567938316 | low |
| GSE57338_GSM1379851_con | 0 | 5.804533 | 7.536982 | 6.158656 | 6.044668 | 5.054619 | 0.892640797 | high |
| GSE57338_GSM1379855_con | 0 | 7.232146 | 7.731633 | 6.643068 | 7.716098 | 5.700008 | 0.88920261 | high |
| GSE57338_GSM1379863_con | 0 | 7.145138 | 7.281483 | 7.538903 | 7.052878 | 5.754536 | 0.95257558 | high |
| GSE57338_GSM1379898_con | 0 | 7.795925 | 7.512679 | 7.00946 | 7.659227 | 5.251559 | 0.734517803 | low |
| GSE57338_GSM1379904_con | 0 | 7.901445 | 7.390449 | 6.532573 | 8.449934 | 4.949405 | 0.750564316 | low |
| GSE57338_GSM1379912_con | 0 | 7.592608 | 8.048183 | 6.271656 | 7.9567 | 5.283519 | 0.599196101 | low |
| GSE57338_GSM1379924_con | 0 | 6.638581 | 6.905775 | 6.251201 | 5.672174 | 4.719916 | 0.624497217 | low |
| GSE57338_GSM1379961_con | 0 | 7.694745 | 7.370222 | 6.326934 | 7.654571 | 5.212365 | 0.665885713 | low |
| GSE57338_GSM1379972_con | 0 | 7.626691 | 7.2093 | 5.850647 | 7.647803 | 4.945906 | 0.568035546 | low |
| GSE57338_GSM1379981_con | 0 | 8.136023 | 6.60619 | 6.85681 | 6.822092 | 5.731123 | 0.703495066 | low |
| GSE57338_GSM1379982_con | 0 | 5.428583 | 6.285667 | 6.786445 | 5.719422 | 6.413674 | 0.997989045 | high |
| GSE57338_GSM1379984_con | 0 | 7.031672 | 6.285667 | 6.626876 | 6.869381 | 5.431515 | 0.955164189 | high |
| GSE57338_GSM1379986_con | 0 | 7.627445 | 7.302848 | 5.874992 | 8.192111 | 4.609483 | 0.612790601 | low |
| GSE57338_GSM1379991_con | 0 | 7.46951 | 7.224148 | 6.494444 | 7.283686 | 5.742937 | 0.837277154 | high |
| GSE57338_GSM1380123_con | 0 | 6.284814 | 6.914643 | 6.392979 | 6.801429 | 4.963724 | 0.951051686 | high |
| GSE76701_GSM2035927_con | 0 | 7.582667 | 8.029023 | 6.307977 | 8.413572 | 5.342834 | 0.748188291 | low |
| GSE1869_GSM33092_treat | 1 | 6.179269 | 6.641098 | 5.74925 | 6.881153 | 5.237881 | 0.964183328 | high |
| GSE1869_GSM33094_treat | 1 | 6.285778 | 6.635471 | 7.102339 | 6.797079 | 5.774413 | 0.99255385 | high |
| GSE1869_GSM33096_treat | 1 | 6.549344 | 7.526409 | 5.759503 | 6.998025 | 4.318464 | 0.632901418 | low |
| GSE1869_GSM33114_treat | 1 | 6.25771 | 7.029214 | 7.028259 | 7.222575 | 4.64382 | 0.974821463 | high |
| GSE1869_GSM33116_treat | 1 | 6.618238 | 6.591423 | 6.031645 | 6.797079 | 5.212161 | 0.931181258 | high |
| GSE1869_GSM33117_treat | 1 | 6.379435 | 6.805063 | 6.900405 | 7.61099 | 5.291831 | 0.991230174 | high |
| GSE1869_GSM33118_treat | 1 | 6.521574 | 7.170641 | 7.368699 | 6.025226 | 5.49569 | 0.940559605 | high |
| GSE1869_GSM33119_treat | 1 | 6.396922 | 6.918805 | 6.751801 | 7.26917 | 5.254616 | 0.98115087 | high |
| GSE1869_GSM33120_treat | 1 | 6.774907 | 6.373542 | 6.404689 | 6.874769 | 5.000759 | 0.942540408 | high |
| GSE1869_GSM33121_treat | 1 | 6.755639 | 6.699693 | 6.509333 | 6.518267 | 4.543658 | 0.832499073 | high |
| GSE5406_GSM123569_treat | 1 | 6.639404 | 7.394953 | 7.287979 | 6.419633 | 5.585397 | 0.937803024 | high |
| GSE5406_GSM123570_treat | 1 | 8.392773 | 6.954123 | 6.735709 | 8.481527 | 5.177948 | 0.757544521 | low |
| GSE5406_GSM123518_treat | 1 | 6.284087 | 6.89349 | 6.634875 | 6.844105 | 5.104484 | 0.96881818 | high |
| GSE5406_GSM123519_treat | 1 | 7.233734 | 7.221929 | 6.873793 | 7.267175 | 5.01005 | 0.844561194 | high |
| GSE5406_GSM123520_treat | 1 | 6.139622 | 7.375921 | 6.756262 | 7.310025 | 5.068515 | 0.976851639 | high |
| GSE5406_GSM123521_treat | 1 | 7.763928 | 6.808337 | 7.591997 | 7.370257 | 6.218537 | 0.963290711 | high |
| GSE5406_GSM123525_treat | 1 | 6.541264 | 7.501165 | 6.401899 | 7.358009 | 4.791327 | 0.896883023 | high |
| GSE5406_GSM123527_treat | 1 | 6.945125 | 7.561264 | 7.285735 | 6.814689 | 4.64856 | 0.790459087 | high |
| GSE5406_GSM123529_treat | 1 | 8.253761 | 6.902864 | 7.112818 | 8.077125 | 7.381118 | 0.977091804 | high |
| GSE5406_GSM123696_treat | 1 | 6.5384 | 7.800487 | 6.73984 | 6.554495 | 4.758482 | 0.755103254 | low |
| GSE5406_GSM123531_treat | 1 | 7.378485 | 8.056581 | 7.513665 | 7.482892 | 5.14933 | 0.794183316 | high |
| GSE5406_GSM123532_treat | 1 | 9.281557 | 6.903255 | 6.734922 | 9.067289 | 5.792478 | 0.675051963 | low |
| GSE5406_GSM123702_treat | 1 | 7.014488 | 7.275803 | 6.988517 | 7.100522 | 4.761892 | 0.848455472 | high |
| GSE5406_GSM123539_treat | 1 | 6.848499 | 7.244423 | 6.429741 | 5.930757 | 4.992842 | 0.602141552 | low |
| GSE5406_GSM123543_treat | 1 | 6.237844 | 6.745857 | 6.70914 | 5.89975 | 4.877867 | 0.919227052 | high |
| GSE5406_GSM123544_treat | 1 | 6.908269 | 6.647155 | 6.340571 | 6.362518 | 5.11499 | 0.834878344 | high |
| GSE5406_GSM123545_treat | 1 | 6.170161 | 7.237589 | 6.232282 | 6.598253 | 4.54893 | 0.87423123 | high |
| GSE5406_GSM123546_treat | 1 | 6.813581 | 7.083021 | 6.959138 | 6.76457 | 4.943732 | 0.896014517 | high |
| GSE5406_GSM123697_treat | 1 | 6.333564 | 7.31309 | 6.896998 | 7.262216 | 5.14933 | 0.973238645 | high |
| GSE5406_GSM123573_treat | 1 | 6.842647 | 6.363259 | 6.226614 | 6.53129 | 5.355695 | 0.922013763 | high |
| GSE5406_GSM123574_treat | 1 | 6.631114 | 6.846141 | 6.534635 | 6.695575 | 4.96295 | 0.913425176 | high |
| GSE5406_GSM123576_treat | 1 | 5.827823 | 6.945005 | 6.268468 | 7.507085 | 4.92938 | 0.9894943 | high |
| GSE5406_GSM123577_treat | 1 | 6.047103 | 6.35984 | 6.23689 | 6.305849 | 4.956277 | 0.968468613 | high |
| GSE5406_GSM123579_treat | 1 | 6.607299 | 6.993434 | 6.384547 | 7.124232 | 4.52342 | 0.891123011 | high |
| GSE5406_GSM123580_treat | 1 | 5.944584 | 7.415972 | 6.662345 | 6.714383 | 5.351482 | 0.973324357 | high |
| GSE5406_GSM123581_treat | 1 | 6.633807 | 5.634428 | 6.488034 | 6.381656 | 5.333328 | 0.980160286 | high |
| GSE5406_GSM123583_treat | 1 | 6.164168 | 6.407524 | 6.711492 | 6.836709 | 4.964195 | 0.985633323 | high |
| GSE5406_GSM123584_treat | 1 | 6.291536 | 6.851451 | 6.417783 | 6.633683 | 5.185606 | 0.956846901 | high |
| GSE5406_GSM123585_treat | 1 | 6.312394 | 7.228825 | 6.774287 | 6.335429 | 5.335391 | 0.938544411 | high |
| GSE5406_GSM123698_treat | 1 | 6.996631 | 6.938984 | 6.609491 | 6.170955 | 5.159906 | 0.758969566 | low |
| GSE5406_GSM123594_treat | 1 | 7.755131 | 7.355108 | 7.608447 | 7.169801 | 5.274587 | 0.792584069 | high |
| GSE5406_GSM123595_treat | 1 | 6.400237 | 7.297768 | 6.978987 | 7.256987 | 5.277101 | 0.975597536 | high |
| GSE5406_GSM123596_treat | 1 | 6.810659 | 7.044011 | 6.6999 | 6.854842 | 4.780682 | 0.868337959 | high |
| GSE5406_GSM123599_treat | 1 | 6.347418 | 7.24643 | 6.878328 | 6.630983 | 5.394808 | 0.958839579 | high |
| GSE5406_GSM123602_treat | 1 | 6.113928 | 7.323528 | 6.591147 | 6.653298 | 4.188276 | 0.878112289 | high |
| GSE5406_GSM123604_treat | 1 | 6.67761 | 7.875714 | 6.814991 | 6.93503 | 5.230613 | 0.855122518 | high |
| GSE5406_GSM123606_treat | 1 | 6.854904 | 6.804655 | 7.479624 | 7.075459 | 5.040905 | 0.968194749 | high |
| GSE5406_GSM123699_treat | 1 | 6.142143 | 6.63723 | 5.726492 | 5.662764 | 5.089859 | 0.851612342 | high |
| GSE5406_GSM123607_treat | 1 | 6.687361 | 7.426023 | 6.260752 | 6.411386 | 5.228591 | 0.762747913 | low |
| GSE5406_GSM123608_treat | 1 | 7.09192 | 7.494144 | 6.968432 | 7.491905 | 5.186918 | 0.898615553 | high |
| GSE5406_GSM123609_treat | 1 | 6.815092 | 7.464159 | 6.738857 | 6.732192 | 4.904816 | 0.79566924 | high |
| GSE5406_GSM123614_treat | 1 | 6.661944 | 7.09129 | 6.690413 | 7.191994 | 5.504046 | 0.964415674 | high |
| GSE5406_GSM123619_treat | 1 | 6.860113 | 7.303049 | 6.323509 | 6.381291 | 5.732016 | 0.823339782 | high |
| GSE5406_GSM123621_treat | 1 | 6.957727 | 7.491229 | 6.485817 | 7.39954 | 5.202556 | 0.869115842 | high |
| GSE5406_GSM123623_treat | 1 | 5.72421 | 6.79405 | 6.16738 | 6.452829 | 4.72186 | 0.967090191 | high |
| GSE5406_GSM123624_treat | 1 | 7.320503 | 7.953905 | 7.488075 | 7.141291 | 4.787376 | 0.68631152 | low |
| GSE5406_GSM123625_treat | 1 | 6.558658 | 7.741339 | 5.889511 | 6.840423 | 5.256493 | 0.768009519 | high |
| GSE5406_GSM123700_treat | 1 | 6.023014 | 6.443447 | 5.424462 | 5.651779 | 4.960285 | 0.8573217 | high |
| GSE5406_GSM123626_treat | 1 | 6.964728 | 6.40518 | 6.742361 | 7.112752 | 5.201859 | 0.960738971 | high |
| GSE5406_GSM123629_treat | 1 | 7.353616 | 7.251204 | 6.705767 | 7.566034 | 5.452422 | 0.888200403 | high |
| GSE5406_GSM123630_treat | 1 | 6.926251 | 7.571359 | 6.884628 | 6.428026 | 5.773057 | 0.846635236 | high |
| GSE5406_GSM123634_treat | 1 | 6.361087 | 7.57604 | 5.947496 | 6.328504 | 4.526849 | 0.618466501 | low |
| GSE5406_GSM123637_treat | 1 | 7.155355 | 7.050269 | 6.57365 | 7.247116 | 5.325275 | 0.890454154 | high |
| GSE5406_GSM123638_treat | 1 | 6.109821 | 7.795464 | 6.564023 | 6.830422 | 5.783333 | 0.963402718 | high |
| GSE5406_GSM123706_treat | 1 | 6.531843 | 7.727112 | 7.027893 | 8.874251 | 5.398775 | 0.993029337 | high |
| GSE5406_GSM123708_treat | 1 | 5.967213 | 8.268841 | 6.05729 | 6.141513 | 4.848225 | 0.652100644 | low |
| GSE5406_GSM123701_treat | 1 | 7.015256 | 7.111146 | 6.414439 | 6.735853 | 5.302585 | 0.820085741 | high |
| GSE5406_GSM123710_treat | 1 | 7.727727 | 6.777079 | 6.013519 | 7.898672 | 5.526466 | 0.846613393 | high |
| GSE5406_GSM123712_treat | 1 | 6.350164 | 5.787219 | 6.897967 | 8.110925 | 4.897947 | 0.998117544 | high |
| GSE5406_GSM123715_treat | 1 | 6.495175 | 6.941526 | 6.570758 | 7.644776 | 5.534521 | 0.986462709 | high |
| GSE5406_GSM123655_treat | 1 | 7.04504 | 6.807016 | 6.289717 | 6.614812 | 5.758417 | 0.88826638 | high |
| GSE5406_GSM123656_treat | 1 | 6.345422 | 6.830967 | 5.990378 | 7.255042 | 5.300654 | 0.969346161 | high |
| GSE5406_GSM123659_treat | 1 | 7.53417 | 6.89044 | 6.256141 | 6.804185 | 5.572582 | 0.721165502 | low |
| GSE5406_GSM123722_treat | 1 | 7.145811 | 7.986654 | 6.824379 | 6.874034 | 4.96483 | 0.579146312 | low |
| GSE5406_GSM123660_treat | 1 | 6.286044 | 7.155364 | 5.652818 | 6.44426 | 4.990775 | 0.821705689 | high |
| GSE5406_GSM123664_treat | 1 | 5.942878 | 7.562654 | 6.520526 | 7.083866 | 4.314215 | 0.930442457 | high |
| GSE5406_GSM123665_treat | 1 | 6.658034 | 7.372748 | 6.467358 | 7.6154 | 5.555167 | 0.96375006 | high |
| GSE5406_GSM123666_treat | 1 | 7.480797 | 6.974069 | 5.983777 | 6.605875 | 6.022077 | 0.717336176 | low |
| GSE5406_GSM123669_treat | 1 | 6.089492 | 6.810304 | 5.693959 | 7.178638 | 4.866292 | 0.959192899 | high |
| GSE5406_GSM123671_treat | 1 | 7.303507 | 6.365851 | 6.512555 | 6.335087 | 5.73295 | 0.877956203 | high |
| GSE5406_GSM123672_treat | 1 | 6.967638 | 7.447892 | 6.1531 | 7.051419 | 6.043799 | 0.890167809 | high |
| GSE5406_GSM123673_treat | 1 | 8.019978 | 6.789357 | 6.727775 | 8.590273 | 5.636602 | 0.939016105 | high |
| GSE5406_GSM123674_treat | 1 | 6.914889 | 6.805069 | 6.557324 | 6.748848 | 5.392714 | 0.914756966 | high |
| GSE5406_GSM123676_treat | 1 | 6.128298 | 6.642132 | 6.919897 | 7.443587 | 5.323323 | 0.995082187 | high |
| GSE5406_GSM123723_treat | 1 | 5.744387 | 6.487957 | 5.987428 | 5.523217 | 4.645949 | 0.914790521 | high |
| GSE5406_GSM123678_treat | 1 | 6.702579 | 7.001918 | 6.274615 | 6.552923 | 5.673508 | 0.912601511 | high |
| GSE5406_GSM123679_treat | 1 | 6.652486 | 7.311004 | 7.220084 | 7.298773 | 5.078325 | 0.961790467 | high |
| GSE5406_GSM123684_treat | 1 | 6.615604 | 7.74311 | 6.687863 | 7.324878 | 4.577273 | 0.84673625 | high |
| GSE5406_GSM123685_treat | 1 | 6.398302 | 7.291127 | 6.870607 | 7.216233 | 5.048872 | 0.964438886 | high |
| GSE5406_GSM123690_treat | 1 | 6.473582 | 6.651765 | 6.900829 | 6.601445 | 5.866776 | 0.984534142 | high |
| GSE5406_GSM123691_treat | 1 | 6.879034 | 6.730746 | 6.601462 | 5.914135 | 4.926071 | 0.749268079 | low |
| GSE5406_GSM123692_treat | 1 | 7.536124 | 7.976469 | 6.24327 | 7.54892 | 5.60171 | 0.608657483 | low |
| GSE5406_GSM123694_treat | 1 | 6.12771 | 6.715961 | 5.966514 | 5.708822 | 5.268672 | 0.896475119 | high |
| GSE5406_GSM123548_treat | 1 | 6.965438 | 7.683975 | 6.371105 | 7.2707 | 4.999918 | 0.758371283 | low |
| GSE5406_GSM123549_treat | 1 | 6.171292 | 6.35601 | 6.062674 | 6.883571 | 5.614953 | 0.987823347 | high |
| GSE5406_GSM123552_treat | 1 | 6.31503 | 6.796707 | 6.473109 | 6.349201 | 4.82551 | 0.920656405 | high |
| GSE5406_GSM123553_treat | 1 | 6.854904 | 7.643415 | 6.62333 | 6.899114 | 5.288526 | 0.823763253 | high |
| GSE5406_GSM123556_treat | 1 | 6.858627 | 7.089372 | 6.553782 | 6.326883 | 5.388551 | 0.833710231 | high |
| GSE5406_GSM123561_treat | 1 | 6.814317 | 7.335564 | 6.724544 | 6.654526 | 5.488419 | 0.885839874 | high |
| GSE5406_GSM123562_treat | 1 | 7.214278 | 7.019389 | 6.675633 | 7.038217 | 4.749914 | 0.776982681 | high |
| GSE5406_GSM123567_treat | 1 | 6.547238 | 6.538569 | 6.766983 | 6.862909 | 5.283844 | 0.97587034 | high |
| GSE5406_GSM123695_treat | 1 | 6.125824 | 6.781179 | 5.350587 | 5.636764 | 4.857198 | 0.719643571 | low |
| GSE5406_GSM123726_treat | 1 | 6.465333 | 7.192418 | 6.532298 | 7.646118 | 5.17279 | 0.973397757 | high |
| GSE16499_GSM414657_treat | 1 | 6.104929 | 6.83926 | 6.137491 | 6.742983 | 5.276869 | 0.969233284 | high |
| GSE16499_GSM414658_treat | 1 | 6.300321 | 6.538742 | 7.077898 | 6.844542 | 5.783219 | 0.993501177 | high |
| GSE16499_GSM414659_treat | 1 | 6.02076 | 7.175767 | 6.827035 | 6.566188 | 5.098251 | 0.969693693 | high |
| GSE16499_GSM414660_treat | 1 | 8.33714 | 6.753098 | 7.085361 | 6.370906 | 6.527394 | 0.691318087 | low |
| GSE16499_GSM414661_treat | 1 | 6.802962 | 6.527297 | 7.714996 | 8.71741 | 6.19488 | 0.999326808 | high |
| GSE16499_GSM414662_treat | 1 | 5.902342 | 6.595012 | 6.676214 | 6.478164 | 5.1909 | 0.986718242 | high |
| GSE16499_GSM414663_treat | 1 | 5.838551 | 6.911954 | 6.127407 | 6.100178 | 5.107691 | 0.950247339 | high |
| GSE16499_GSM414664_treat | 1 | 5.360754 | 6.538742 | 6.50541 | 5.832287 | 5.170419 | 0.989425884 | high |
| GSE16499_GSM414665_treat | 1 | 4.750732 | 6.541473 | 6.210941 | 6.615639 | 4.809464 | 0.997627003 | high |
| GSE16499_GSM414666_treat | 1 | 6.352143 | 6.238868 | 6.7127 | 6.75804 | 5.700413 | 0.9915035 | high |
| GSE16499_GSM414667_treat | 1 | 6.205229 | 6.635238 | 6.866708 | 7.340226 | 5.259689 | 0.992732731 | high |
| GSE16499_GSM414668_treat | 1 | 6.672872 | 6.692121 | 6.344412 | 6.374397 | 4.659832 | 0.828522269 | high |
| GSE16499_GSM414669_treat | 1 | 6.015809 | 7.021354 | 6.926564 | 6.305268 | 5.24371 | 0.973937798 | high |
| GSE16499_GSM414670_treat | 1 | 6.558462 | 6.577852 | 7.642383 | 7.044259 | 6.045703 | 0.996024456 | high |
| GSE16499_GSM414671_treat | 1 | 6.262794 | 6.308077 | 6.670911 | 6.829765 | 5.214049 | 0.987564048 | high |
| GSE42955_GSM1053920_treat | 1 | 6.840784 | 7.806003 | 6.200156 | 8.150855 | 4.954782 | 0.887875766 | high |
| GSE42955_GSM1053921_treat | 1 | 6.342663 | 5.932963 | 7.273447 | 6.460225 | 5.431667 | 0.99385524 | high |
| GSE42955_GSM1053923_treat | 1 | 5.838466 | 7.682227 | 6.531042 | 5.584964 | 4.512513 | 0.750070182 | low |
| GSE42955_GSM1053927_treat | 1 | 6.444847 | 6.407104 | 6.878123 | 7.346058 | 5.606154 | 0.994001461 | high |
| GSE42955_GSM1053928_treat | 1 | 6.16324 | 6.879195 | 6.989974 | 6.097322 | 5.180033 | 0.962636267 | high |
| GSE42955_GSM1053930_treat | 1 | 6.888915 | 7.760761 | 5.722632 | 7.593301 | 6.060181 | 0.889451098 | high |
| GSE42955_GSM1053931_treat | 1 | 6.373955 | 6.670933 | 6.155394 | 6.415322 | 4.809404 | 0.903849184 | high |
| GSE42955_GSM1053916_treat | 1 | 6.327588 | 6.641854 | 6.212069 | 7.99251 | 5.282922 | 0.991917262 | high |
| GSE42955_GSM1053932_treat | 1 | 6.646389 | 7.145226 | 6.797477 | 7.187733 | 5.733307 | 0.97363078 | high |
| GSE42955_GSM1053934_treat | 1 | 7.017515 | 8.380701 | 6.363867 | 7.521725 | 4.913546 | 0.582234913 | low |
| GSE42955_GSM1053936_treat | 1 | 5.759935 | 6.293471 | 7.262652 | 6.127961 | 5.201197 | 0.994322595 | high |
| GSE52601_GSM1272372_treat | 1 | 5.693236 | 6.300115 | 6.32117 | 6.340322 | 5.529535 | 0.993109926 | high |
| GSE52601_GSM1272374_treat | 1 | 5.070325 | 6.429857 | 6.460731 | 6.801146 | 4.899776 | 0.99774341 | high |
| GSE52601_GSM1272376_treat | 1 | 5.456826 | 6.852845 | 7.167934 | 6.493099 | 6.636821 | 0.999040718 | high |
| GSE52601_GSM1272380_treat | 1 | 5.808574 | 7.335859 | 6.476456 | 6.140198 | 4.819082 | 0.927144429 | high |
| GSE52601_GSM1272386_treat | 1 | 7.716341 | 7.213911 | 6.729129 | 7.720081 | 5.326886 | 0.809250786 | high |
| GSE52601_GSM1272387_treat | 1 | 7.404799 | 6.620476 | 6.785611 | 6.594137 | 4.799916 | 0.732670328 | low |
| GSE52601_GSM1272391_treat | 1 | 7.747962 | 6.661839 | 7.323414 | 6.511251 | 5.210979 | 0.754460739 | low |
| GSE57338_GSM1379813_treat | 1 | 5.017428 | 6.904577 | 6.572636 | 5.72302 | 5.696688 | 0.994902984 | high |
| GSE57338_GSM1379814_treat | 1 | 5.927105 | 5.631056 | 6.753933 | 4.607889 | 5.59162 | 0.977999954 | high |
| GSE57338_GSM1379815_treat | 1 | 7.390429 | 4.667244 | 6.092926 | 9.653182 | 6.087397 | 0.999628636 | high |
| GSE57338_GSM1379819_treat | 1 | 6.481042 | 6.575166 | 6.411266 | 6.676202 | 5.393371 | 0.965583748 | high |
| GSE57338_GSM1379821_treat | 1 | 5.258224 | 6.853806 | 6.319805 | 6.341172 | 5.162213 | 0.991451764 | high |
| GSE57338_GSM1379822_treat | 1 | 5.035967 | 6.888705 | 6.398075 | 6.326983 | 5.280477 | 0.99528905 | high |
| GSE57338_GSM1379823_treat | 1 | 5.774692 | 7.005879 | 6.744322 | 6.661894 | 5.403876 | 0.989570764 | high |
| GSE57338_GSM1379824_treat | 1 | 6.36983 | 6.262259 | 7.375375 | 6.700497 | 5.520671 | 0.993848166 | high |
| GSE57338_GSM1379825_treat | 1 | 4.79213 | 6.330809 | 6.755418 | 6.621773 | 5.427387 | 0.999411655 | high |
| GSE57338_GSM1379826_treat | 1 | 6.583449 | 6.406637 | 7.108243 | 7.167713 | 5.294642 | 0.989077943 | high |
| GSE57338_GSM1379828_treat | 1 | 4.884783 | 6.458812 | 6.546405 | 5.58002 | 5.355381 | 0.996172782 | high |
| GSE57338_GSM1379829_treat | 1 | 5.958363 | 7.328952 | 7.215583 | 6.877338 | 5.3139 | 0.9876272 | high |
| GSE57338_GSM1379928_treat | 1 | 7.450816 | 6.210629 | 8.367434 | 6.071821 | 6.011588 | 0.97558121 | high |
| GSE57338_GSM1379930_treat | 1 | 6.446955 | 6.891157 | 7.4968 | 7.515939 | 5.620917 | 0.995064971 | high |
| GSE57338_GSM1379933_treat | 1 | 7.214881 | 7.456845 | 6.831051 | 7.869643 | 4.702238 | 0.854287062 | high |
| GSE57338_GSM1379938_treat | 1 | 7.442961 | 6.024234 | 6.653966 | 7.306284 | 5.958589 | 0.974984532 | high |
| GSE57338_GSM1379943_treat | 1 | 6.074907 | 6.162047 | 7.29159 | 6.041995 | 5.387816 | 0.991956952 | high |
| GSE57338_GSM1379945_treat | 1 | 5.198684 | 6.58636 | 6.41147 | 6.046664 | 5.860259 | 0.996711574 | high |
| GSE57338_GSM1379946_treat | 1 | 8.308573 | 5.868764 | 7.401478 | 6.818037 | 5.636471 | 0.869277986 | high |
| GSE57338_GSM1379948_treat | 1 | 6.513026 | 5.543444 | 7.991336 | 6.23575 | 6.096676 | 0.998335764 | high |
| GSE57338_GSM1379949_treat | 1 | 4.982515 | 6.13314 | 6.661053 | 6.359122 | 5.793075 | 0.999318452 | high |
| GSE57338_GSM1379951_treat | 1 | 7.318589 | 5.363788 | 8.298852 | 5.318742 | 6.67728 | 0.991929968 | high |
| GSE57338_GSM1379952_treat | 1 | 8.014052 | 5.792176 | 7.459295 | 8.399576 | 5.56597 | 0.988538332 | high |
| GSE57338_GSM1379953_treat | 1 | 6.559333 | 5.008931 | 7.105416 | 6.480578 | 5.415042 | 0.996589603 | high |
| GSE57338_GSM1379958_treat | 1 | 6.055817 | 6.858966 | 7.038009 | 6.604172 | 5.50878 | 0.988995606 | high |
| GSE57338_GSM1379959_treat | 1 | 6.422336 | 7.279612 | 7.404569 | 6.198311 | 5.179622 | 0.93850822 | high |
| GSE57338_GSM1379960_treat | 1 | 5.07641 | 6.943354 | 6.655742 | 5.439069 | 5.479895 | 0.990188782 | high |
| GSE57338_GSM1379964_treat | 1 | 6.038038 | 7.277378 | 6.887329 | 5.955403 | 5.743739 | 0.965359951 | high |
| GSE57338_GSM1379992_treat | 1 | 7.030274 | 6.288919 | 7.338304 | 7.724911 | 5.665568 | 0.993475994 | high |
| GSE57338_GSM1379997_treat | 1 | 6.387289 | 6.672519 | 7.346816 | 6.56261 | 5.685475 | 0.989045764 | high |
| GSE57338_GSM1379998_treat | 1 | 6.119572 | 6.711005 | 7.010289 | 6.896042 | 4.936544 | 0.986107044 | high |
| GSE57338_GSM1379999_treat | 1 | 6.572158 | 6.597812 | 6.53048 | 6.496883 | 5.853639 | 0.971172322 | high |
| GSE57338_GSM1380000_treat | 1 | 6.094074 | 5.78583 | 7.486936 | 6.641966 | 5.996629 | 0.998911417 | high |
| GSE57338_GSM1380001_treat | 1 | 6.191064 | 6.045103 | 7.814381 | 6.492289 | 6.479176 | 0.999042907 | high |
| GSE57338_GSM1380002_treat | 1 | 6.02438 | 6.415073 | 7.323812 | 6.258972 | 5.492725 | 0.993261185 | high |
| GSE57338_GSM1380004_treat | 1 | 5.834026 | 5.493896 | 7.467125 | 6.557195 | 5.669178 | 0.999299361 | high |
| GSE57338_GSM1380005_treat | 1 | 5.96344 | 6.728705 | 7.377989 | 6.622092 | 5.710801 | 0.995640336 | high |
| GSE57338_GSM1380018_treat | 1 | 7.936564 | 6.696158 | 7.526963 | 7.7482 | 6.530279 | 0.97761696 | high |
| GSE57338_GSM1380019_treat | 1 | 5.388188 | 6.934405 | 6.397813 | 6.456104 | 5.461821 | 0.992732618 | high |
| GSE57338_GSM1380024_treat | 1 | 6.605706 | 6.763261 | 6.897281 | 6.944458 | 5.290961 | 0.971291487 | high |
| GSE57338_GSM1380030_treat | 1 | 5.397604 | 6.525808 | 6.305732 | 6.278517 | 5.394573 | 0.993651211 | high |
| GSE57338_GSM1380040_treat | 1 | 5.344197 | 7.004899 | 6.873653 | 5.275058 | 4.959438 | 0.969059098 | high |
| GSE57338_GSM1380041_treat | 1 | 7.338348 | 6.339917 | 7.341613 | 6.769136 | 5.999676 | 0.972197512 | high |
| GSE57338_GSM1380042_treat | 1 | 5.877133 | 6.800924 | 6.693332 | 5.592356 | 5.144354 | 0.95298693 | high |
| GSE57338_GSM1380043_treat | 1 | 5.140074 | 6.699735 | 6.613472 | 5.409523 | 6.114346 | 0.995523515 | high |
| GSE57338_GSM1380044_treat | 1 | 5.024888 | 6.392962 | 6.822954 | 5.471351 | 5.446938 | 0.996319536 | high |
| GSE57338_GSM1380045_treat | 1 | 4.609795 | 6.56639 | 6.263634 | 5.046558 | 5.466003 | 0.99448957 | high |
| GSE57338_GSM1380047_treat | 1 | 5.780481 | 6.827299 | 6.666984 | 6.447272 | 5.013055 | 0.982379277 | high |
| GSE57338_GSM1380049_treat | 1 | 5.075303 | 6.746874 | 6.625114 | 5.836481 | 5.026378 | 0.992034801 | high |
| GSE57338_GSM1380051_treat | 1 | 7.456213 | 7.139726 | 7.448762 | 7.201504 | 6.006279 | 0.947927033 | high |
| GSE57338_GSM1380052_treat | 1 | 5.167707 | 7.396511 | 6.607178 | 6.118235 | 4.881306 | 0.981176461 | high |
| GSE57338_GSM1380054_treat | 1 | 5.389743 | 6.919122 | 6.703617 | 6.936561 | 5.217264 | 0.996097411 | high |
| GSE57338_GSM1380055_treat | 1 | 5.053279 | 6.79045 | 6.999944 | 6.005002 | 5.286784 | 0.996540437 | high |
| GSE57338_GSM1380057_treat | 1 | 5.704914 | 6.299347 | 6.644807 | 6.695988 | 5.790378 | 0.997450499 | high |
| GSE57338_GSM1380060_treat | 1 | 5.902585 | 6.273986 | 7.049537 | 6.534382 | 5.45985 | 0.995710827 | high |
| GSE57338_GSM1380061_treat | 1 | 6.233619 | 6.557736 | 6.679116 | 7.273462 | 4.938186 | 0.987246459 | high |
| GSE57338_GSM1380063_treat | 1 | 6.240646 | 7.099132 | 7.090385 | 6.955222 | 6.038842 | 0.992194445 | high |
| GSE57338_GSM1380064_treat | 1 | 6.801592 | 6.06643 | 7.229393 | 6.420117 | 6.513545 | 0.993574976 | high |
| GSE57338_GSM1380065_treat | 1 | 5.703673 | 7.098094 | 6.684293 | 5.96514 | 4.810296 | 0.955011 | high |
| GSE57338_GSM1380066_treat | 1 | 5.177784 | 5.960732 | 6.675676 | 6.554824 | 5.316077 | 0.998936972 | high |
| GSE57338_GSM1380067_treat | 1 | 5.639946 | 6.718889 | 6.845819 | 6.14141 | 4.743065 | 0.981543339 | high |
| GSE57338_GSM1380069_treat | 1 | 8.0917 | 7.024252 | 6.503109 | 8.785312 | 5.453552 | 0.890450994 | high |
| GSE57338_GSM1380070_treat | 1 | 5.202525 | 6.442682 | 6.830766 | 6.351506 | 5.71916 | 0.998522074 | high |
| GSE57338_GSM1380071_treat | 1 | 5.792887 | 6.962366 | 6.365691 | 6.756218 | 5.070684 | 0.981141879 | high |
| GSE57338_GSM1380074_treat | 1 | 6.423958 | 5.364031 | 6.237198 | 5.191271 | 6.045488 | 0.977869543 | high |
| GSE57338_GSM1380076_treat | 1 | 4.875233 | 6.400751 | 6.212203 | 4.173607 | 5.224505 | 0.971193236 | high |
| GSE57338_GSM1380079_treat | 1 | 5.370104 | 6.31454 | 6.41147 | 6.261557 | 5.789292 | 0.997246087 | high |
| GSE57338_GSM1380081_treat | 1 | 6.998494 | 7.14983 | 6.272209 | 7.420966 | 5.197982 | 0.887085025 | high |
| GSE57338_GSM1380086_treat | 1 | 6.505625 | 7.171153 | 7.106272 | 7.017978 | 5.638964 | 0.979499981 | high |
| GSE57338_GSM1380087_treat | 1 | 5.367108 | 6.732291 | 6.973054 | 6.543496 | 5.473803 | 0.997284525 | high |
| GSE57338_GSM1380089_treat | 1 | 5.970582 | 5.413276 | 6.54488 | 7.290407 | 5.971663 | 0.99937167 | high |
| GSE57338_GSM1380090_treat | 1 | 5.062447 | 6.006137 | 6.294283 | 4.370297 | 5.680116 | 0.988433355 | high |
| GSE57338_GSM1380091_treat | 1 | 5.518536 | 7.122332 | 6.337323 | 7.20589 | 5.00287 | 0.991351541 | high |
| GSE57338_GSM1380092_treat | 1 | 4.803318 | 5.89972 | 6.753222 | 6.246659 | 6.082399 | 0.999733578 | high |
| GSE57338_GSM1380093_treat | 1 | 6.195798 | 6.994915 | 6.609748 | 6.55054 | 4.691161 | 0.935175766 | high |
| GSE57338_GSM1380097_treat | 1 | 5.38898 | 6.482375 | 6.777471 | 5.841789 | 5.331315 | 0.993348447 | high |
| GSE57338_GSM1380100_treat | 1 | 5.027459 | 6.502965 | 7.131627 | 6.044963 | 6.203802 | 0.99929177 | high |
| GSE57338_GSM1380101_treat | 1 | 6.291051 | 6.578597 | 5.818832 | 6.258427 | 5.15386 | 0.915547245 | high |
| GSE57338_GSM1380104_treat | 1 | 5.389227 | 5.822013 | 6.706195 | 5.587851 | 5.26473 | 0.995583165 | high |
| GSE57338_GSM1380105_treat | 1 | 5.864223 | 6.889005 | 6.514111 | 6.192013 | 5.828524 | 0.985109197 | high |
| GSE57338_GSM1380106_treat | 1 | 5.475637 | 6.752573 | 7.329735 | 5.303538 | 5.231542 | 0.986302555 | high |
| GSE57338_GSM1380107_treat | 1 | 5.206583 | 6.676327 | 6.699253 | 6.866899 | 4.967059 | 0.997206294 | high |
| GSE57338_GSM1380108_treat | 1 | 6.041047 | 7.005569 | 6.609214 | 6.564456 | 5.038477 | 0.966533796 | high |
| GSE57338_GSM1380109_treat | 1 | 5.555838 | 6.660533 | 6.776496 | 6.694777 | 4.899881 | 0.993166659 | high |
| GSE57338_GSM1380110_treat | 1 | 5.53894 | 7.179905 | 6.718575 | 6.685287 | 5.252363 | 0.990546038 | high |
| GSE57338_GSM1380111_treat | 1 | 5.948851 | 7.053194 | 6.452574 | 6.635072 | 5.537058 | 0.981232297 | high |
| GSE57338_GSM1380112_treat | 1 | 5.413848 | 6.779219 | 6.684293 | 7.098157 | 5.949829 | 0.998687124 | high |
| GSE57338_GSM1380113_treat | 1 | 5.499336 | 6.880612 | 7.101944 | 6.167324 | 6.699234 | 0.998406935 | high |
| GSE57338_GSM1380114_treat | 1 | 5.311868 | 6.284038 | 6.722103 | 5.923439 | 5.523439 | 0.996566264 | high |
| GSE57338_GSM1380115_treat | 1 | 6.205114 | 7.155189 | 6.675926 | 7.267739 | 5.478233 | 0.985294868 | high |
| GSE57338_GSM1380116_treat | 1 | 6.468634 | 6.632343 | 7.515368 | 6.46734 | 6.110655 | 0.992594383 | high |
| GSE57338_GSM1380117_treat | 1 | 6.623078 | 6.144657 | 7.791543 | 6.69694 | 6.25154 | 0.997290167 | high |
| GSE57338_GSM1380119_treat | 1 | 6.732403 | 6.699994 | 7.262509 | 7.742791 | 5.835019 | 0.99470109 | high |
| GSE57338_GSM1380120_treat | 1 | 4.958461 | 6.611371 | 6.942419 | 6.419788 | 5.578477 | 0.998930202 | high |
| GSE76701_GSM2035928_treat | 1 | 6.559169 | 5.888543 | 7.135063 | 6.48828 | 6.337089 | 0.996198158 | high |
| GSE76701_GSM2035929_treat | 1 | 6.34101 | 7.393327 | 6.404552 | 5.665991 | 5.093521 | 0.737922667 | low |
| GSE76701_GSM2035930_treat | 1 | 5.655092 | 6.775698 | 7.25126 | 6.176281 | 5.43263 | 0.99364214 | high |
| GSE76701_GSM2035931_treat | 1 | 5.521164 | 6.296268 | 6.617735 | 6.498638 | 5.043103 | 0.994968625 | high |
| GSE21610_GSM545670_treat | 1 | 7.860297 | 7.218426 | 6.774596 | 7.443453 | 4.735686 | 0.555764129 | low |
| GSE21610_GSM545672_treat | 1 | 5.528592 | 6.527726 | 5.799131 | 5.13828 | 5.496332 | 0.954273701 | high |
| GSE21610_GSM545675_treat | 1 | 6.058343 | 5.913868 | 6.10999 | 6.356483 | 5.647182 | 0.99059563 | high |
| GSE21610_GSM545676_treat | 1 | 6.338208 | 8.13977 | 6.778994 | 6.229446 | 5.53107 | 0.829538075 | high |
| GSE21610_GSM545680_treat | 1 | 7.604018 | 6.490624 | 6.830646 | 7.043459 | 5.450748 | 0.885315222 | high |
| GSE21610_GSM545687_treat | 1 | 6.709451 | 7.819007 | 6.407304 | 7.026786 | 5.085777 | 0.791555967 | high |
| GSE21610_GSM545691_treat | 1 | 5.721262 | 6.975164 | 7.214902 | 7.045555 | 6.157353 | 0.998393123 | high |
| GSE21610_GSM545694_treat | 1 | 6.787255 | 7.219097 | 6.806976 | 6.401881 | 5.368473 | 0.870802446 | high |
| GSE21610_GSM545700_treat | 1 | 4.093765 | 6.479189 | 6.31744 | 6.406837 | 5.117898 | 0.999530247 | high |
| GSE21610_GSM545702_treat | 1 | 6.546194 | 7.116229 | 6.532919 | 6.39416 | 5.02446 | 0.868528339 | high |
| GSE21610_GSM545705_treat | 1 | 8.033027 | 5.921792 | 6.659344 | 8.958674 | 4.758263 | 0.962890706 | high |
| GSE21610_GSM545706_treat | 1 | 7.64474 | 8.235504 | 6.926523 | 7.71957 | 5.545965 | 0.667954023 | low |
| GSE21610_GSM545709_treat | 1 | 6.377954 | 7.515155 | 6.869251 | 7.168765 | 5.321322 | 0.96399175 | high |
| GSE21610_GSM545717_treat | 1 | 8.332621 | 7.875586 | 6.759522 | 8.576839 | 5.623949 | 0.665011468 | low |
| GSE21610_GSM545721_treat | 1 | 6.109731 | 6.568777 | 7.110995 | 7.72123 | 5.544993 | 0.997973414 | high |
| GSE21610_GSM545724_treat | 1 | 7.484948 | 7.650674 | 6.460469 | 7.645136 | 4.826103 | 0.61378195 | low |
| GSE1869_GSM33108_con | 0 | 7.396571 | 8.591936 | 6.085945 | 6.996332 | 4.596699 | 0.123494628 | low |
| GSE1869_GSM33109_con | 0 | 8.91308 | 10.13321 | 5.858639 | 8.49286 | 5.109937 | 0.006869919 | low |
| GSE1869_GSM33110_con | 0 | 9.990799 | 9.169398 | 5.837961 | 9.102546 | 5.087071 | 0.005143601 | low |
| GSE1869_GSM33111_con | 0 | 7.941293 | 8.808888 | 5.756144 | 6.884331 | 5.544173 | 0.057274421 | low |
| GSE1869_GSM33112_con | 0 | 9.55991 | 7.640562 | 6.277885 | 8.698885 | 5.161984 | 0.086083915 | low |
| GSE1869_GSM33113_con | 0 | 7.921601 | 7.551015 | 6.206215 | 6.721802 | 5.024777 | 0.191751865 | low |
| GSE5406_GSM123640_con | 0 | 8.823995 | 9.900699 | 5.183758 | 7.901491 | 4.275076 | 0.001164744 | low |
| GSE5406_GSM123641_con | 0 | 8.917346 | 8.041226 | 5.328341 | 7.902603 | 4.889372 | 0.023356217 | low |
| GSE5406_GSM123643_con | 0 | 9.041957 | 8.509401 | 5.422461 | 8.52674 | 4.035133 | 0.009180268 | low |
| GSE5406_GSM123644_con | 0 | 11.411163 | 8.947694 | 5.149385 | 8.892598 | 4.526849 | 8.02E-05 | low |
| GSE5406_GSM123645_con | 0 | 11.306247 | 10.985447 | 5.29927 | 8.698492 | 4.232873 | 4.77E-06 | low |
| GSE5406_GSM123646_con | 0 | 8.868472 | 9.655362 | 5.817491 | 8.207056 | 4.966907 | 0.008191568 | low |
| GSE5406_GSM123647_con | 0 | 9.659166 | 8.684747 | 5.34232 | 8.485376 | 4.499985 | 0.002997661 | low |
| GSE5406_GSM123649_con | 0 | 9.36388 | 8.940244 | 4.818908 | 8.402234 | 4.084458 | 0.001369633 | low |
| GSE5406_GSM123536_con | 0 | 10.586404 | 8.311641 | 5.545126 | 8.16592 | 4.497045 | 0.000602456 | low |
| GSE5406_GSM123575_con | 0 | 7.278719 | 10.112811 | 5.828132 | 6.85914 | 4.253725 | 0.011285142 | low |
| GSE5406_GSM123582_con | 0 | 9.167903 | 9.160044 | 5.603952 | 7.567401 | 4.236202 | 0.001462381 | low |
| GSE5406_GSM123705_con | 0 | 9.773984 | 10.742605 | 5.582455 | 8.871237 | 4.935172 | 0.000527913 | low |
| GSE5406_GSM123639_con | 0 | 10.66219 | 9.583558 | 4.798565 | 8.56524 | 3.92362 | 4.12E-05 | low |
| GSE5406_GSM123709_con | 0 | 10.204109 | 8.901398 | 5.438516 | 8.233704 | 4.730887 | 0.000773106 | low |
| GSE5406_GSM123727_con | 0 | 9.433604 | 11.080115 | 4.781058 | 8.624014 | 4.316876 | 0.0001196 | low |
| GSE16499_GSM414642_con | 0 | 9.545333 | 8.637686 | 6.285482 | 8.085588 | 4.339826 | 0.005335146 | low |
| GSE16499_GSM414643_con | 0 | 8.546048 | 8.947187 | 5.724615 | 8.545134 | 4.452285 | 0.030483157 | low |
| GSE16499_GSM414644_con | 0 | 7.601278 | 8.039466 | 6.372825 | 7.368171 | 4.624089 | 0.286600433 | low |
| GSE16499_GSM414645_con | 0 | 6.615769 | 8.309178 | 6.086041 | 5.746463 | 4.970289 | 0.255858696 | low |
| GSE16499_GSM414646_con | 0 | 8.874065 | 8.789402 | 5.455802 | 7.797882 | 4.798126 | 0.008925268 | low |
| GSE16499_GSM414647_con | 0 | 8.542182 | 8.272205 | 6.124428 | 7.317172 | 4.984244 | 0.044455569 | low |
| GSE16499_GSM414648_con | 0 | 9.046379 | 9.522289 | 6.212414 | 7.984072 | 4.997307 | 0.007903315 | low |
| GSE16499_GSM414649_con | 0 | 8.530143 | 8.382089 | 6.016882 | 8.384446 | 4.479913 | 0.071249613 | low |
| GSE16499_GSM414651_con | 0 | 9.053656 | 8.009996 | 5.827088 | 7.784642 | 5.329498 | 0.04116608 | low |
| GSE16499_GSM414652_con | 0 | 8.415729 | 8.289731 | 5.786768 | 8.006829 | 5.047006 | 0.092991658 | low |
| GSE16499_GSM414653_con | 0 | 9.194774 | 8.264349 | 4.925214 | 8.976934 | 4.534013 | 0.0163284 | low |
| GSE16499_GSM414654_con | 0 | 8.330744 | 8.491369 | 5.553666 | 7.745249 | 4.291447 | 0.023739031 | low |
| GSE16499_GSM414655_con | 0 | 9.021447 | 8.826895 | 5.368288 | 9.615541 | 4.444366 | 0.033175002 | low |
| GSE42955_GSM1053922_con | 0 | 9.742253 | 8.19825 | 5.084759 | 7.172965 | 4.180303 | 0.000551433 | low |
| GSE42955_GSM1053929_con | 0 | 8.252904 | 7.288477 | 5.678995 | 7.368634 | 4.765024 | 0.140358017 | low |
| GSE42955_GSM1053939_con | 0 | 8.844232 | 8.563214 | 6.167981 | 8.142184 | 5.194683 | 0.056341309 | low |
| GSE42955_GSM1053940_con | 0 | 9.489785 | 8.971601 | 5.847837 | 8.989739 | 4.582018 | 0.00951952 | low |
| GSE42955_GSM1053942_con | 0 | 9.535161 | 9.596294 | 6.393462 | 8.276723 | 5.268395 | 0.005958289 | low |
| GSE52601_GSM1272369_con | 0 | 9.060281 | 7.390409 | 6.196846 | 8.388578 | 4.485504 | 0.101037576 | low |
| GSE52601_GSM1272378_con | 0 | 9.205991 | 8.841157 | 5.625887 | 7.847251 | 4.646207 | 0.004509466 | low |
| GSE52601_GSM1272382_con | 0 | 8.97677 | 9.947389 | 5.394701 | 9.162814 | 4.666538 | 0.006668467 | low |
| GSE52601_GSM1272388_con | 0 | 7.79476 | 8.96626 | 5.320796 | 8.437372 | 4.756069 | 0.105486937 | low |
| GSE57338_GSM1379830_con | 0 | 7.260417 | 9.289858 | 5.290451 | 7.512978 | 4.433992 | 0.050685411 | low |
| GSE57338_GSM1379831_con | 0 | 8.443021 | 7.896562 | 6.632524 | 8.096686 | 5.281806 | 0.347190419 | low |
| GSE57338_GSM1379832_con | 0 | 7.733754 | 9.240272 | 5.478449 | 7.679342 | 4.318921 | 0.027095527 | low |
| GSE57338_GSM1379833_con | 0 | 8.784407 | 8.056156 | 6.047256 | 8.241669 | 4.357613 | 0.050227298 | low |
| GSE57338_GSM1379834_con | 0 | 7.61787 | 9.665476 | 4.93466 | 7.649615 | 4.139012 | 0.00939459 | low |
| GSE57338_GSM1379836_con | 0 | 8.840166 | 8.916858 | 5.898086 | 8.198976 | 4.850292 | 0.021100043 | low |
| GSE57338_GSM1379837_con | 0 | 8.539955 | 8.044245 | 5.843692 | 7.607296 | 4.545267 | 0.040206386 | low |
| GSE57338_GSM1379838_con | 0 | 8.334379 | 8.452553 | 5.929239 | 8.133951 | 4.893733 | 0.10023202 | low |
| GSE57338_GSM1379839_con | 0 | 7.912396 | 7.934618 | 5.761448 | 8.051656 | 4.25459 | 0.167166828 | low |
| GSE57338_GSM1379840_con | 0 | 8.474015 | 9.170099 | 5.361487 | 7.903115 | 4.900952 | 0.014240051 | low |
| GSE57338_GSM1379841_con | 0 | 8.722185 | 8.781992 | 5.508511 | 8.755856 | 4.74045 | 0.036920214 | low |
| GSE57338_GSM1379843_con | 0 | 8.013546 | 7.93337 | 6.216294 | 8.267557 | 4.462206 | 0.292259076 | low |
| GSE57338_GSM1379844_con | 0 | 7.705224 | 8.57126 | 5.51991 | 7.485026 | 5.231542 | 0.134247636 | low |
| GSE57338_GSM1379846_con | 0 | 8.404723 | 9.085646 | 5.739784 | 7.809213 | 4.153697 | 0.010694465 | low |
| GSE57338_GSM1379847_con | 0 | 7.345199 | 7.75656 | 5.427963 | 7.478898 | 4.880776 | 0.36749794 | low |
| GSE57338_GSM1379848_con | 0 | 6.821414 | 7.617192 | 6.090293 | 6.328607 | 4.624827 | 0.432605695 | low |
| GSE57338_GSM1379849_con | 0 | 7.098572 | 7.369835 | 5.908345 | 6.487319 | 4.82869 | 0.427240766 | low |
| GSE57338_GSM1379850_con | 0 | 8.155066 | 8.745793 | 5.236662 | 7.994933 | 4.583183 | 0.032693747 | low |
| GSE57338_GSM1379852_con | 0 | 7.970845 | 8.161341 | 5.974487 | 7.644636 | 4.632336 | 0.131665662 | low |
| GSE57338_GSM1379853_con | 0 | 7.080458 | 7.847127 | 6.006595 | 7.534379 | 4.331678 | 0.482629107 | low |
| GSE57338_GSM1379854_con | 0 | 7.960045 | 8.508136 | 6.071351 | 8.07279 | 4.374735 | 0.120591259 | low |
| GSE57338_GSM1379856_con | 0 | 7.449642 | 8.873593 | 5.959767 | 7.687421 | 4.347904 | 0.118583054 | low |
| GSE57338_GSM1379857_con | 0 | 8.574749 | 7.822091 | 5.56379 | 8.34638 | 4.651375 | 0.09606633 | low |
| GSE57338_GSM1379858_con | 0 | 8.746607 | 7.28188 | 5.422434 | 8.890577 | 4.623763 | 0.194787036 | low |
| GSE57338_GSM1379859_con | 0 | 8.840166 | 8.749687 | 5.591027 | 8.512386 | 4.535367 | 0.020029656 | low |
| GSE57338_GSM1379860_con | 0 | 8.550376 | 9.130608 | 6.145494 | 7.78279 | 4.500531 | 0.015619126 | low |
| GSE57338_GSM1379861_con | 0 | 9.115431 | 7.965707 | 5.979082 | 7.924658 | 4.426695 | 0.02037797 | low |
| GSE57338_GSM1379862_con | 0 | 8.697401 | 8.54017 | 5.707171 | 7.693067 | 4.773053 | 0.019322815 | low |
| GSE57338_GSM1379864_con | 0 | 8.907924 | 8.515029 | 5.319416 | 7.648767 | 4.588395 | 0.006956252 | low |
| GSE57338_GSM1379865_con | 0 | 7.656956 | 9.190301 | 4.95977 | 7.342863 | 4.111717 | 0.01107816 | low |
| GSE57338_GSM1379866_con | 0 | 8.807796 | 8.371227 | 5.857497 | 8.003597 | 4.968755 | 0.038856871 | low |
| GSE57338_GSM1379867_con | 0 | 7.914153 | 9.588359 | 5.406039 | 7.718847 | 4.206333 | 0.010471023 | low |
| GSE57338_GSM1379868_con | 0 | 7.614529 | 8.438503 | 5.537627 | 7.400154 | 4.958357 | 0.132123728 | low |
| GSE57338_GSM1379869_con | 0 | 8.455689 | 9.059479 | 5.51753 | 7.81071 | 4.799328 | 0.015963142 | low |
| GSE57338_GSM1379870_con | 0 | 9.403915 | 8.294556 | 5.798457 | 9.154744 | 4.515859 | 0.028879664 | low |
| GSE57338_GSM1379871_con | 0 | 7.424496 | 7.287065 | 6.317708 | 6.286522 | 5.218699 | 0.43248752 | low |
| GSE57338_GSM1379872_con | 0 | 9.176416 | 8.696936 | 5.871444 | 7.961264 | 4.636483 | 0.008305953 | low |
| GSE57338_GSM1379873_con | 0 | 8.058582 | 7.754906 | 5.850097 | 7.747687 | 4.323853 | 0.133242624 | low |
| GSE57338_GSM1379874_con | 0 | 9.119159 | 9.208618 | 5.127135 | 7.850937 | 4.867076 | 0.002613931 | low |
| GSE57338_GSM1379875_con | 0 | 8.112001 | 9.406989 | 5.688479 | 8.391208 | 4.220368 | 0.025841191 | low |
| GSE57338_GSM1379876_con | 0 | 7.786299 | 8.462171 | 5.597169 | 8.057664 | 4.558071 | 0.134915101 | low |
| GSE57338_GSM1379877_con | 0 | 8.526485 | 8.715452 | 5.800584 | 8.086615 | 4.592552 | 0.031147039 | low |
| GSE57338_GSM1379878_con | 0 | 9.102451 | 7.76933 | 6.471002 | 8.368066 | 4.801029 | 0.101745319 | low |
| GSE57338_GSM1379880_con | 0 | 9.150165 | 7.192411 | 6.142586 | 8.26544 | 4.554412 | 0.096237462 | low |
| GSE57338_GSM1379881_con | 0 | 8.441696 | 8.248208 | 6.3132 | 8.003597 | 4.804668 | 0.116852444 | low |
| GSE57338_GSM1379882_con | 0 | 8.930464 | 8.002036 | 5.700254 | 7.9764 | 5.452732 | 0.06602823 | low |
| GSE57338_GSM1379883_con | 0 | 8.804799 | 8.631177 | 6.194095 | 8.273223 | 4.657016 | 0.038338699 | low |
| GSE57338_GSM1379884_con | 0 | 9.312415 | 8.719181 | 5.92179 | 8.143685 | 5.007679 | 0.011822065 | low |
| GSE57338_GSM1379885_con | 0 | 8.890338 | 9.078091 | 6.636336 | 8.238114 | 4.842681 | 0.0328326 | low |
| GSE57338_GSM1379886_con | 0 | 8.299763 | 8.73116 | 5.869891 | 7.657316 | 4.905354 | 0.043283807 | low |
| GSE57338_GSM1379887_con | 0 | 8.89761 | 7.693515 | 5.522021 | 8.464427 | 4.376913 | 0.050408244 | low |
| GSE57338_GSM1379888_con | 0 | 8.025305 | 8.371227 | 6.102673 | 8.109362 | 4.887827 | 0.211188612 | low |
| GSE57338_GSM1379889_con | 0 | 8.279409 | 8.495715 | 6.330266 | 8.411606 | 4.658718 | 0.159051376 | low |
| GSE57338_GSM1379890_con | 0 | 8.244592 | 8.619811 | 6.491543 | 7.829767 | 4.696018 | 0.09574216 | low |
| GSE57338_GSM1379891_con | 0 | 8.080199 | 7.460601 | 6.485028 | 7.682174 | 4.866509 | 0.400376771 | low |
| GSE57338_GSM1379892_con | 0 | 8.19583 | 7.693995 | 6.021924 | 7.276338 | 4.796892 | 0.123776739 | low |
| GSE57338_GSM1379893_con | 0 | 8.337532 | 7.786389 | 6.130699 | 8.106656 | 4.559441 | 0.177774919 | low |
| GSE57338_GSM1379894_con | 0 | 9.219982 | 7.523622 | 7.25127 | 8.2967 | 4.830726 | 0.200232517 | low |
| GSE57338_GSM1379895_con | 0 | 8.865785 | 8.758804 | 5.959505 | 7.98362 | 4.368931 | 0.012156027 | low |
| GSE57338_GSM1379896_con | 0 | 8.420606 | 8.865948 | 5.777564 | 7.815999 | 4.637071 | 0.023885859 | low |
| GSE57338_GSM1379897_con | 0 | 9.000819 | 8.880833 | 5.520935 | 8.064384 | 4.6133 | 0.007336408 | low |
| GSE57338_GSM1379899_con | 0 | 8.212823 | 7.021228 | 5.881013 | 8.108723 | 4.44148 | 0.341989566 | low |
| GSE57338_GSM1379900_con | 0 | 8.89871 | 8.43263 | 6.080665 | 8.530074 | 4.832322 | 0.058583173 | low |
| GSE57338_GSM1379901_con | 0 | 8.237003 | 8.69451 | 5.341507 | 8.130661 | 4.527711 | 0.036097483 | low |
| GSE57338_GSM1379902_con | 0 | 7.727318 | 8.625584 | 5.553115 | 7.268873 | 4.113124 | 0.032104793 | low |
| GSE57338_GSM1379903_con | 0 | 8.709298 | 7.950511 | 6.292234 | 8.109362 | 4.469392 | 0.079920432 | low |
| GSE57338_GSM1379905_con | 0 | 7.105934 | 8.05554 | 6.407341 | 6.874786 | 4.679972 | 0.400208497 | low |
| GSE57338_GSM1379906_con | 0 | 8.476767 | 7.596262 | 6.835407 | 7.290785 | 5.630775 | 0.333459343 | low |
| GSE57338_GSM1379907_con | 0 | 9.815232 | 7.779378 | 6.433928 | 9.139829 | 4.983255 | 0.070250054 | low |
| GSE57338_GSM1379908_con | 0 | 8.89871 | 8.097213 | 5.750272 | 8.383312 | 4.273607 | 0.030970986 | low |
| GSE57338_GSM1379909_con | 0 | 8.217281 | 7.986459 | 6.069263 | 8.09609 | 4.577161 | 0.1683677 | low |
| GSE57338_GSM1379910_con | 0 | 8.827231 | 8.228382 | 6.25323 | 8.67137 | 4.789298 | 0.111672462 | low |
| GSE57338_GSM1379911_con | 0 | 8.466323 | 9.202243 | 5.807737 | 8.429028 | 4.69684 | 0.031716297 | low |
| GSE57338_GSM1379913_con | 0 | 8.203721 | 8.295465 | 5.752354 | 6.991173 | 4.286291 | 0.019731144 | low |
| GSE57338_GSM1379914_con | 0 | 7.887179 | 8.006702 | 6.046445 | 8.170439 | 4.93519 | 0.379079713 | low |
| GSE57338_GSM1379915_con | 0 | 7.562921 | 9.142136 | 6.037957 | 7.513736 | 5.019084 | 0.119832292 | low |
| GSE57338_GSM1379918_con | 0 | 8.362198 | 8.159153 | 5.638448 | 6.286795 | 4.528524 | 0.008640416 | low |
| GSE57338_GSM1379919_con | 0 | 8.402822 | 8.096466 | 5.993382 | 8.622368 | 5.234306 | 0.296215552 | low |
| GSE57338_GSM1379920_con | 0 | 7.948727 | 8.402547 | 6.228468 | 7.862472 | 4.516158 | 0.145634092 | low |
| GSE57338_GSM1379921_con | 0 | 8.588736 | 7.8533 | 7.018202 | 8.623189 | 5.053801 | 0.471157378 | low |
| GSE57338_GSM1379922_con | 0 | 8.597912 | 8.920027 | 6.184004 | 8.449136 | 4.793432 | 0.055979552 | low |
| GSE57338_GSM1379923_con | 0 | 7.545564 | 7.804293 | 5.855378 | 7.59018 | 4.514754 | 0.297924663 | low |
| GSE57338_GSM1379925_con | 0 | 8.352655 | 8.590278 | 5.676253 | 8.032153 | 4.580612 | 0.042386672 | low |
| GSE57338_GSM1379926_con | 0 | 8.786197 | 8.189705 | 5.149207 | 8.696902 | 4.47836 | 0.034487227 | low |
| GSE57338_GSM1379927_con | 0 | 9.316165 | 8.251336 | 5.588124 | 8.539702 | 4.524725 | 0.014683172 | low |
| GSE57338_GSM1379962_con | 0 | 8.235195 | 8.60615 | 6.095293 | 7.821067 | 4.608348 | 0.062749997 | low |
| GSE57338_GSM1379965_con | 0 | 8.619905 | 8.486362 | 5.194661 | 7.964928 | 5.063747 | 0.027435317 | low |
| GSE57338_GSM1379966_con | 0 | 8.168638 | 7.190974 | 5.987302 | 7.308168 | 4.549525 | 0.180634867 | low |
| GSE57338_GSM1379967_con | 0 | 8.957309 | 9.098425 | 5.837732 | 8.051656 | 4.319198 | 0.005947691 | low |
| GSE57338_GSM1379968_con | 0 | 9.058673 | 9.044883 | 5.803912 | 8.288871 | 4.910627 | 0.012444232 | low |
| GSE57338_GSM1379969_con | 0 | 10.24838 | 8.851989 | 5.57667 | 8.284006 | 4.640123 | 0.000830115 | low |
| GSE57338_GSM1379970_con | 0 | 8.255284 | 8.128321 | 5.507976 | 8.130661 | 4.59444 | 0.086818162 | low |
| GSE57338_GSM1379971_con | 0 | 7.266915 | 7.763084 | 6.730106 | 6.332493 | 5.102319 | 0.44326846 | low |
| GSE57338_GSM1379973_con | 0 | 7.217863 | 7.210349 | 6.397813 | 5.446352 | 5.196319 | 0.334283091 | low |
| GSE57338_GSM1379974_con | 0 | 8.467719 | 8.223067 | 5.917367 | 7.915468 | 4.921643 | 0.08221075 | low |
| GSE57338_GSM1379975_con | 0 | 7.639798 | 7.910543 | 6.689316 | 7.658282 | 4.57379 | 0.444712368 | low |
| GSE57338_GSM1379976_con | 0 | 8.528897 | 7.706528 | 6.241802 | 8.033984 | 4.762473 | 0.170772031 | low |
| GSE57338_GSM1379977_con | 0 | 8.972829 | 7.930924 | 5.814354 | 7.932087 | 4.518416 | 0.026877706 | low |
| GSE57338_GSM1379978_con | 0 | 8.697401 | 7.650093 | 6.523718 | 8.215777 | 5.385577 | 0.334014091 | low |
| GSE57338_GSM1379979_con | 0 | 7.855176 | 8.404344 | 6.047508 | 7.362873 | 5.023572 | 0.141298628 | low |
| GSE57338_GSM1379980_con | 0 | 7.896307 | 7.845433 | 7.124543 | 6.50928 | 5.521183 | 0.358789613 | low |
| GSE57338_GSM1379983_con | 0 | 8.435512 | 7.76667 | 5.943974 | 7.365511 | 5.350095 | 0.127784462 | low |
| GSE57338_GSM1379985_con | 0 | 9.413084 | 9.259775 | 5.721615 | 8.321198 | 4.660913 | 0.003358248 | low |
| GSE57338_GSM1379987_con | 0 | 8.421875 | 7.926532 | 6.468221 | 8.338732 | 4.742086 | 0.253126656 | low |
| GSE57338_GSM1379988_con | 0 | 8.092748 | 7.477577 | 6.072667 | 7.244017 | 4.796346 | 0.18944917 | low |
| GSE57338_GSM1379989_con | 0 | 8.526485 | 8.503921 | 6.699764 | 7.999642 | 4.045571 | 0.048890447 | low |
| GSE57338_GSM1379990_con | 0 | 7.358449 | 8.02432 | 5.905984 | 6.919478 | 4.782619 | 0.228849931 | low |
| GSE57338_GSM1380008_con | 0 | 8.059631 | 8.651629 | 5.481088 | 8.068693 | 4.235444 | 0.042518048 | low |
| GSE57338_GSM1380010_con | 0 | 7.617506 | 7.424092 | 5.731091 | 7.043168 | 4.986379 | 0.31494324 | low |
| GSE57338_GSM1380011_con | 0 | 7.88443 | 8.398937 | 5.630653 | 7.659227 | 4.360583 | 0.067218266 | low |
| GSE57338_GSM1380012_con | 0 | 7.774929 | 8.600519 | 5.350981 | 7.969429 | 4.570041 | 0.087180393 | low |
| GSE57338_GSM1380013_con | 0 | 7.559001 | 8.967896 | 5.642936 | 7.402908 | 4.429548 | 0.05140393 | low |
| GSE57338_GSM1380014_con | 0 | 8.436889 | 8.433568 | 5.36624 | 8.003053 | 4.530224 | 0.029816987 | low |
| GSE57338_GSM1380015_con | 0 | 8.800704 | 9.356291 | 5.901205 | 8.067519 | 4.44148 | 0.007263499 | low |
| GSE57338_GSM1380021_con | 0 | 7.370341 | 8.004738 | 5.769694 | 7.644636 | 4.775474 | 0.378534863 | low |
| GSE57338_GSM1380027_con | 0 | 8.693871 | 8.257501 | 5.733976 | 8.06683 | 4.863292 | 0.04802989 | low |
| GSE57338_GSM1380122_con | 0 | 9.048909 | 9.545266 | 5.54749 | 8.742574 | 4.280213 | 0.004520637 | low |
| GSE57338_GSM1380124_con | 0 | 8.719636 | 9.411977 | 6.115169 | 7.641027 | 4.585694 | 0.006945044 | low |
| GSE76701_GSM2035924_con | 0 | 8.0103 | 7.966729 | 5.909047 | 8.303762 | 4.15135 | 0.180155195 | low |
| GSE76701_GSM2035925_con | 0 | 9.257237 | 9.112716 | 5.377867 | 7.979407 | 4.628774 | 0.002571207 | low |
| GSE76701_GSM2035926_con | 0 | 9.31588 | 8.770603 | 5.856084 | 8.419303 | 4.609885 | 0.009329153 | low |
| GSE21610_GSM545657_con | 0 | 8.138938 | 8.151509 | 5.927243 | 7.156045 | 5.199786 | 0.096610472 | low |
| GSE21610_GSM545658_con | 0 | 8.703715 | 7.382845 | 5.612506 | 7.670358 | 4.749631 | 0.070016566 | low |
| GSE21610_GSM545659_con | 0 | 8.580396 | 7.540307 | 6.466396 | 6.9962 | 5.086898 | 0.106064077 | low |
| GSE21610_GSM545660_con | 0 | 8.595478 | 9.176917 | 6.651313 | 8.014501 | 3.866151 | 0.014704315 | low |
| GSE21610_GSM545661_con | 0 | 8.073363 | 8.262607 | 5.799131 | 7.385578 | 5.19202 | 0.108435562 | low |
| GSE21610_GSM545662_con | 0 | 7.732551 | 8.414021 | 5.466834 | 7.373398 | 5.11563 | 0.116510404 | low |
| GSE21610_GSM545663_con | 0 | 8.24033 | 8.897929 | 5.489966 | 7.774402 | 3.603594 | 0.007962118 | low |
| GSE21610_GSM545664_con | 0 | 8.587861 | 8.572086 | 5.753987 | 8.112727 | 4.795588 | 0.040131183 | low |
| GSE5406_GSM123526_treat | 1 | 9.154659 | 7.564457 | 6.234281 | 9.409494 | 4.851785 | 0.276178419 | low |
| GSE5406_GSM123703_treat | 1 | 9.955841 | 8.106572 | 4.99122 | 9.972386 | 4.637639 | 0.016258229 | low |
| GSE5406_GSM123534_treat | 1 | 9.22979 | 7.495811 | 6.21232 | 10.084161 | 5.021133 | 0.482908111 | low |
| GSE5406_GSM123598_treat | 1 | 7.259112 | 9.900699 | 6.595955 | 7.888812 | 4.428678 | 0.120149516 | low |
| GSE5406_GSM123600_treat | 1 | 6.746711 | 8.410078 | 6.377862 | 6.528809 | 5.110223 | 0.474670929 | low |
| GSE5406_GSM123605_treat | 1 | 8.786202 | 7.481577 | 6.463666 | 7.115588 | 4.827677 | 0.06793457 | low |
| GSE5406_GSM123615_treat | 1 | 8.079375 | 9.181295 | 6.337146 | 7.522906 | 4.66124 | 0.039680762 | low |
| GSE5406_GSM123632_treat | 1 | 7.769546 | 8.301118 | 6.620873 | 7.395412 | 5.691041 | 0.456900875 | low |
| GSE5406_GSM123707_treat | 1 | 8.624826 | 9.760073 | 4.897902 | 10.024822 | 3.927121 | 0.013396167 | low |
| GSE5406_GSM123642_treat | 1 | 10.318765 | 6.910181 | 5.065328 | 10.344415 | 6.265844 | 0.262177843 | low |
| GSE5406_GSM123648_treat | 1 | 7.386295 | 9.165142 | 6.808217 | 7.17495 | 4.52342 | 0.137565647 | low |
| GSE5406_GSM123713_treat | 1 | 9.314197 | 7.533299 | 4.955371 | 9.682812 | 5.416669 | 0.170656988 | low |
| GSE5406_GSM123716_treat | 1 | 7.485151 | 7.974374 | 6.110405 | 7.32725 | 5.261596 | 0.447579212 | low |
| GSE5406_GSM123680_treat | 1 | 7.282768 | 7.202688 | 6.05729 | 6.706097 | 4.593111 | 0.424925641 | low |
| GSE5406_GSM123558_treat | 1 | 8.273259 | 8.18543 | 6.978987 | 7.775934 | 5.299957 | 0.337259362 | low |
| GSE42955_GSM1053914_treat | 1 | 6.994544 | 7.687731 | 6.432231 | 6.486812 | 4.67313 | 0.464550331 | low |
| GSE52601_GSM1272379_treat | 1 | 7.243632 | 7.529295 | 6.367989 | 6.410096 | 5.169518 | 0.482514207 | low |
| GSE57338_GSM1380007_treat | 1 | 8.244592 | 8.256694 | 6.130175 | 7.352545 | 5.114963 | 0.095599101 | low |
| GSE21610_GSM545679_treat | 1 | 7.567695 | 7.144714 | 5.171661 | 7.131661 | 5.274388 | 0.3894203 | low |
| GSE21610_GSM545710_treat | 1 | 7.892647 | 8.432677 | 6.659344 | 7.647961 | 3.978953 | 0.109084347 | low |
